# Inflammation-centered neurovascular–immune–metabolic remodeling in ischemic stroke: stage-dependent mechanisms, regulated cell death, and therapeutic translation

**DOI:** 10.3389/fimmu.2026.1918327

**Published:** 2026-09-03

**Authors:** Linjing Song, Zijin Sun, Xuhui Zhang, Guojiao Shang, Shanyu Du, Fafeng Cheng, Wenxiu Xu

**Affiliations:** 1 First School of Clinical Medicine, Beijing University of Chinese Medicine, Beijing, China; 2 School of Chinese Medicine, Beijing University of Chinese Medicine, Beijing, China; 3 School of Medicine, University of Health and Rehabilitation Sciences, Shandong, China; 4 School of Life Sciences and Health, University of Health and Rehabilitation Sciences, Shandong, China

**Keywords:** immunometabolism, ischemic stroke, neuroinflammation, neurovascular unit, regulated cell death

## Abstract

Ischemic stroke evolves beyond arterial occlusion through an inflammation-centered network linking neurovascular dysfunction, immune remodeling, metabolic reprogramming, and regulated cell death. These interactions are organized across the hyperacute, acute, subacute, and chronic phases. Hyperacute energy failure, excitotoxicity, thromboinflammation, pericyte contraction, and capillary stalling can sustain microcirculatory no-reflow despite recanalization. Acute injury is characterized by blood–brain barrier disruption, innate immune amplification, mitochondrial stress, as well as ferroptotic, necroptotic, pyroptotic, and proposed cuproptotic pathways. Subacute recovery involves debris clearance, immune resolution, angiogenesis, metabolic adaptation, and oligodendrocyte-lineage repair, whereas chronic outcomes reflect persistent inflammation, white-matter remodeling, and neural plasticity. Cell-specific metabolism and brain-border and systemic immune–metabolic communication further shape injury and recovery. Experimental evidence is distinguished from findings in human blood, thrombectomy-derived samples, imaging, and brain tissue. Therapeutic translation requires stage-matched interventions compatible with reperfusion and rehabilitation, clinically realistic post-onset dosing, mechanistic biomarkers, and appropriate safety evaluation. This framework links early microvascular rescue with immune resolution, metabolic recovery, and network repair.

## 1 Introduction

Ischemic stroke (IS) remains a major global cause of mortality and long-term disability (1). The Global Burden of Disease 2021 analysis shows that, although age-standardized stroke rates have declined in many settings since 1990, the absolute burden has continued to increase (1, 2). China-specific analyses further illustrate substantial geographic heterogeneity (3).

In current clinical practice, intravenous thrombolysis and mechanical thrombectomy improve outcomes in eligible patients (3), but access and benefit remain constrained by treatment eligibility and time. Reperfusion can also initiate a general ischemia–reperfusion injury cascade involving oxidative, inflammatory, and mitochondrial stress (4). Even after successful large-vessel recanalization, microcirculatory failure may persist: pericyte constriction can contribute to no-reflow (5), while damage-associated molecular patterns (DAMPs) can drive neutrophil extracellular trap (NET) formation, stabilize thrombi, impair thrombolysis, disrupt the blood–brain barrier (BBB), and amplify neuroinflammation (6).

Tissue injury after IS extends far beyond the initial interruption of cerebral blood flow. Instead, it evolves as a progressive and highly interconnected pathological network involving BBB breakdown, oxidative stress, and robust neuroinflammatory activation (7). The infiltration and activation of diverse immune-cell populations within the brain parenchyma are tightly intertwined with disturbances in energy metabolism, collectively shaping the transition from acute injury to chronic remodeling (8). This pronounced clinical and biological heterogeneity, arising from the interplay among vascular pathology, metabolic failure, and immune responses, indicates that strategies focused solely on early vascular recanalization or single-target neuronal protection are insufficient to fully explain or address the complex pathological challenges that emerge after IS.

Against this backdrop, the concept of neurovascular–immune–metabolic remodeling has emerged as an integrative framework for understanding IS. This concept refers to the coordinated and dynamic reorganization of resident cells within the central nervous system (CNS), key structural and functional components of the vascular microenvironment, and peripherally infiltrating immune cells at the transcriptional, metabolic, and spatial levels under ischemic–hypoxic stress (9). Rather than representing a simple aggregation of isolated cellular events, this process reflects a systems-level interaction network in which neuronal tissue injury, vascular barrier disintegration, immune-inflammatory activation, and energy metabolic reprogramming are causally interconnected and functionally integrated.

Within this remodeling framework, the interplay between immune-cell state transitions and cellular metabolism constitutes a central hub that drives disease progression. Ischemia-induced bioenergetic crisis forces profound metabolic rewiring within the cerebral microenvironment. For example, the loss of mitochondrial metabolic flexibility in microglial and macrophage populations, together with enforced shifts in glycolipid metabolic pathways, directly shapes their phenotypic transition between proinflammatory neurotoxicity and tissue repair (10, 11). Concomitantly, extensive intercellular communication networks further amplify this cross-system integration. Astrocytes not only undergo intrinsic metabolic remodeling to regulate BBB homeostasis and the neurotransmitter milieu (12), but also modulate the lipid metabolic trajectories and inflammatory response states of microglia through specific receptor–ligand interactions and paracrine signaling mechanisms (13). Vascular microenvironmental components, including endothelial cells and fibroblasts, likewise participate in the regulation of immune responses and the metabolic microecology through complex multicellular interactions.

Inflammation is not an isolated downstream consequence of cerebral ischemia. It is a dynamic interface through which vascular dysfunction, immune-cell recruitment, glial activation, metabolic stress, and regulated cell death influence one another. Endothelial activation and BBB disruption promote leukocyte and platelet recruitment; activated immune and glial cells reshape local glucose, lactate, lipid, and amino-acid metabolism; and metabolite accumulation, mitochondrial dysfunction, and lipid peroxidation further amplify inflammatory signaling. These processes are strongly time-dependent and may shift from tissue-destructive to reparative functions as stroke evolves.

Accordingly, this review uses inflammation as the organizing axis to integrate three closely connected dimensions of ischemic-stroke biology: neurovascular unit (NVU) and microcirculatory remodeling, local and systemic immune responses, and cell- and substrate-specific metabolic reprogramming. Within this framework, ischemia- and reperfusion-induced disturbances in communication among neurons, glial cells, vascular cells, circulating immune cells, and systemic metabolic organs are considered across the transition from early injury amplification to tissue containment, repair, and functional remodeling. Particular emphasis is placed on temporal and biological context, because the same cell population, metabolite, or inflammatory pathway may exert divergent or even opposing effects during the hyperacute, acute, subacute, and chronic phases. The discussion focuses on convergent mechanisms linking vascular dysfunction, immune activation, metabolic failure, and regulated cell death, rather than on an exhaustive catalogue of signaling molecules or experimental compounds. Findings from experimental models are interpreted alongside available human evidence, with attention to stroke etiology, reperfusion status, aging, and common metabolic comorbidities. This inflammation-centered and temporally resolved framework provides a basis for identifying the principal determinants of injury and recovery, evaluating barriers to clinical translation, and defining therapeutic opportunities that require stage-specific and biologically stratified intervention.

## 2 Stage-dependent evolution of ischemic injury and remodeling

### 2.1 Hyperacute phase: energy failure and injury initiation

During the hyperacute phase of IS, abrupt interruption of cerebral blood flow rapidly deprives the brain of oxygen and glucose, impairing neuronal mitochondrial oxidative phosphorylation and adenosine triphosphate (ATP) production. Loss of ion-pump function then drives membrane depolarization, glutamate release, ionic disequilibrium, and calcium overload. Mechanistic syntheses describe this as a continuous cascade linking bioenergetic failure, excitotoxicity, oxidative stress, regulated cell death, and mitochondrial dysfunction (4, 14). Metabolomic studies likewise connect disturbances in glutamate metabolism and the tricarboxylic acid cycle with oxidative stress, inflammation, and energy failure (15). Excessive activation of N-methyl-D-aspartate receptors (NMDARs) and voltage-dependent calcium channels increases intracellular Ca^2+^, which promotes mitochondrial dysfunction, endoplasmic reticulum stress, reactive oxygen species (ROS) generation, and calcium-dependent injury pathways (16, 17).

As a critical hub for energy metabolism and cell-death regulation, mitochondria exhibit early reductions in membrane potential, impaired bioenergetic capacity, and heightened oxidative stress after ischemic insult (18). When damaged mitochondria are not efficiently removed, mitochondrial dysfunction and oxidative injury can form a self-amplifying pathological loop (19). In addition, aberrant activation of transient receptor potential melastatin channels and uncoupling of neuronal nitric oxide synthase may further aggravate early neuronal injury by promoting calcium overload, ROS generation, mitochondrial damage, and energy loss (20, 21). Thus, hyperacute ischemic injury is not merely a consequence of oxygen deprivation. Rather, it begins with metabolic collapse and rapidly connects ionic disequilibrium, excitotoxicity, calcium overload, and mitochondrial injury, thereby establishing the pathological foundation for subsequent neurovascular–immune–metabolic remodeling.

### 2.2 Acute phase: barrier failure and inflammatory amplification

As ischemia–reperfusion progresses into the acute phase, NVU injury is initially manifested by endothelial damage and loss of barrier stability. The endothelial cell-derived arachidonate 12-lipoxygenase (ALOX12)–12-hydroxyeicosatetraenoic acid (12-HETE) axis is upregulated after cerebral ischemia–reperfusion, impairing tight junction integrity while concomitantly promoting proinflammatory microglial activation and oxidative neuronal injury (22). Overexpression of nicotinamide adenine dinucleotide phosphate oxidase 5 (NOX5) similarly aggravates ischemia-associated neuroinflammation by altering endothelial junctional architecture, increasing vascular permeability, and facilitating immune-cell adhesion and infiltration (23). Thus, acute BBB disruption is not merely a passive structural failure. Rather, it represents a dynamic process driven by endothelial oxidative stress, cytoskeletal remodeling, and degradation of tight junction proteins.

The pathological increase in BBB permeability creates a permissive route for peripheral immune cells to enter the brain parenchyma, thereby establishing a self-reinforcing inflammatory loop with locally activated microglia. Microglial activation, neutrophil infiltration, and the release of proinflammatory cytokines collectively exacerbate BBB breakdown and neuronal death, whereas BBB injury further facilitates immune-cell entry and inflammatory dissemination (24). In patients with acute IS, elevated serum levels of NOD-like receptor family pyrin domain-containing 3 (NLRP3) and occludin are associated with infarct volume, National Institutes of Health Stroke Scale (NIHSS) scores, hemorrhagic transformation, and poor clinical outcomes, suggesting a clinically relevant link between inflammasome activation and tight junction injury (25). Experimental evidence further indicates that serum amyloid A (SAA) signaling can induce inflammation, glial activation, and increased BBB permeability through NLRP3-dependent mechanisms (26).

Inflammatory amplification during the acute phase also involves the gliovascular unit and peripheral thromboinflammatory signals. After ischemic injury, perivascular upregulation of secreted phosphoprotein 1 (SPP1) is accompanied by enhanced microglial synaptic engulfment, aggravated neuroinflammation, and impaired BBB integrity (27). NET formation further disrupts vascular integrity through protease activity, inflammatory signaling, and microvascular obstruction, with translational and experimental studies linking NETs to brain injury, resistance to thrombolysis, and impaired vascular repair (28–30). Fibrinogen can also bind integrin subunit beta 2 (ITGB2) on microglia and activate JAK–STAT signaling, thereby altering microglial function while aggravating BBB disruption and neuroinflammation (31). Collectively, BBB breakdown, immune-cell infiltration, microglial activation, and inflammatory-mediator release form a self-amplifying acute injury cascade.

### 2.3 Subacute phase: immune resolution and repair initiation

Across the acute-to-subacute transition, the inflammatory response undergoes a dynamic transition from acute tissue injury toward reparative remodeling. At this stage, large numbers of peripheral monocytes are recruited into the injured brain and differentiate into MDMs. These cells serve a central clearance function by phagocytosing necrotic neurons and myelin debris, while simultaneously activating lysosomal- and lipid metabolism-associated gene programs that define highly phagocytic Cd68hi/Ctsdhi subsets (32). MDMs also exhibit phenotypic adaptation during the subacute phase, shifting from an early inflammation-associated state toward a microglia-like phenotype. This transition is accompanied by enhanced phagocytic activity and provides a cellular basis for debris removal and inflammatory resolution within injured tissue (33).

Across the acute-to-subacute transition, microglia display marked functional plasticity. Itgb2+ microglial subsets participate in energy metabolism, cell-cycle regulation, angiogenesis, and myelination at days 1, 3, and 7 after ischemic injury, respectively (34). In an interleukin-4 (IL-4)-conditioned mouse transient middle cerebral artery occlusion (MCAO) paradigm, microglial extracellular vesicles (EVs) enriched in miR-23a-5p promoted oligodendrocyte precursor cells (OPCs) proliferation, survival, and differentiation and supported white-matter repair (35). Regulatory T cells (Tregs)-derived osteopontin can strengthen reparative microglial functions through integrin receptors and facilitate OPCs differentiation and white-matter regeneration (36). Local BBB-associated chemokines and neuropeptides, including pituitary adenylate cyclase-activating polypeptide, may likewise favor repair-supporting microglial functions in experimental models (37).

Fibrotic remodeling and vascular regeneration are likewise integral components of subacute repair. In IL-4-conditioned experimental systems, macrophages promoted meningeal-fibroblast proliferation, migration, and extracellular matrix (ECM) production through the PU.1/mTOR axis (38). In a rat MCAO/reperfusion model, IL-4-conditioned macrophages promoted early fibrotic-scar formation through transforming growth factor beta 1 (TGF-β1) and matrix metalloproteinase (MMP)-9, with concurrent angiogenic and functional changes (39). An enriched environment can also reduce microglia-associated proinflammatory cytokine production and support white-matter regeneration (40). These findings show that beneficial inflammatory functions depend on cellular state, timing, and phagocytic competence rather than on a fixed binary macrophage label.

### 2.4 Chronic phase: network reconstruction and functional recovery

During the chronic phase of IS, the neurovascular–immune–metabolic network is progressively reorganized and maintained in a dynamic equilibrium. At this stage, inflammation is not uniformly detrimental; rather, when properly regulated, it contributes to tissue repair and functional restoration. Microglia exhibit diverse phenotypic states during chronic remodeling, and their interactions with neurons, astrocytes, and endothelial cells shape neural plasticity and synaptic reorganization. Through the secretion of soluble mediators and EVs, microglia also regulate the local inflammatory milieu and BBB integrity (41, 42). OPCs acquire an oligodendrogenic state during the chronic phase and contribute to white-matter repair and remyelination, with their function being influenced by local oxygen tension and the vascular microenvironment (43). Endothelial cells likewise display stage-specific differentiation and enhanced anti-inflammatory signaling, thereby regulating vascular homeostasis and promoting microvascular angiogenesis. In parallel, endothelial adhesion molecules selectively guide lymphocyte infiltration and support neurovascular repair (44).

**Figure 1:**
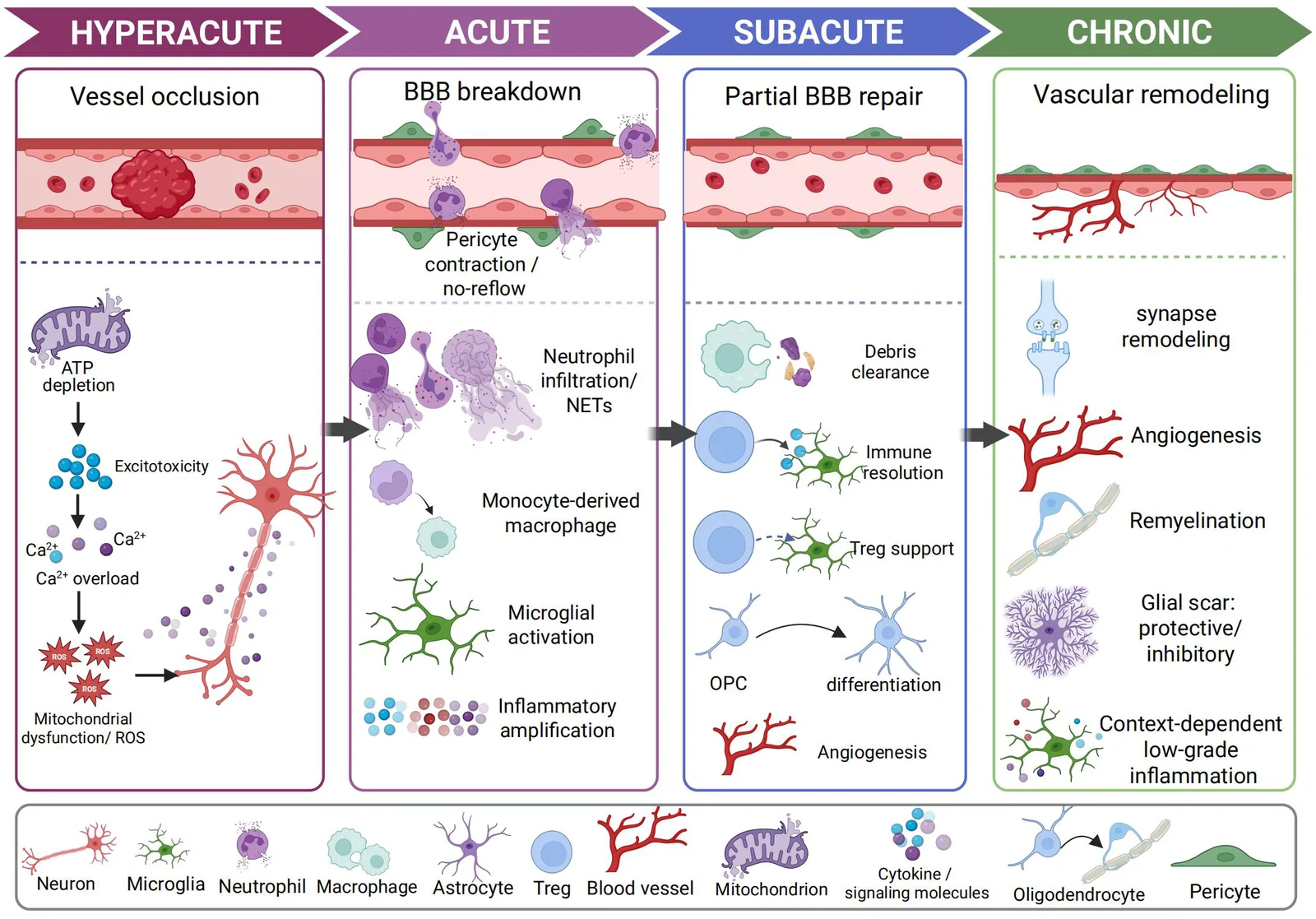
Stage-dependent evolution of neurovascular-immune-metabolic remodeling after ischemic stroke. The four color-coded columns (dark magenta, hyperacute; violet, acute; blue, subacute; and green, chronic) depict the overlapping progression of post-ischemic injury and repair, with gray arrows indicating movement across phases. In the hyperacute phase, vessel occlusion initiates ATP depletion, excitotoxicity, Ca^2+^ overload, mitochondrial dysfunction, ROS production, and neuronal injury. In the acute phase, BBB breakdown, abluminal pericyte contraction, and microcirculatory no-reflow accompany neutrophil infiltration and NET formation, recruitment of monocyte-derived macrophages, microglial activation, and inflammatory amplification. In the subacute phase, partial BBB repair occurs alongside macrophage-mediated debris clearance, immune resolution, Treg support, OPC differentiation, and angiogenesis. In the chronic phase, vascular remodeling, synaptic remodeling, angiogenesis, and remyelination coexist with the context-dependent protective or inhibitory effects of the glial scar and persistent low-grade inflammation. Green pericytes are positioned on the abluminal side of the endothelial layer. The branched red microvasculature beneath the parent vessel in the chronic column represents angiogenic growth in the surrounding parenchyma rather than intraluminal sprouting. Dashed horizontal lines visually separate vascular changes above from parenchymal immune, metabolic, and repair processes below. The symbol key identifies neurons, microglia, neutrophils, macrophages, astrocytes, Tregs, blood vessels, mitochondria, cytokines/signaling molecules, oligodendrocytes, and pericytes. ACOD1/IRG1, aconitate decarboxylase 1/immune-responsive gene 1; ATP, adenosine triphosphate; BBB, blood–brain barrier; BDNF, brain-derived neurotrophic factor; Ca^2+^, calcium ion; cfDNA, cell-free DNA; cGAS–STING, cyclic GMP–AMP synthase–stimulator of interferon genes; CH_3_, methyl group; CH25H, cholesterol 25-hydroxylase; Cu^2+^, copper(II) ion; DAMPs, damage-associated molecular patterns; dsDNA, double-stranded DNA; ECM, extracellular matrix; EVs, extracellular vesicles; Fe^2+^, ferrous ion; G6PD, glucose-6-phosphate dehydrogenase; GABA, γ-aminobutyric acid; GDNF, glial cell line-derived neurotrophic factor; GPX4, glutathione peroxidase 4; GSH, glutathione; HT, hemorrhagic transformation; IL, interleukin; IV, intravenous; LNPs, lipid nanoparticles; M1/M2, conventional labels for classically/alternatively activated myeloid states; miRNAs, microRNAs; MMP, matrix metalloproteinase; MMP-9, matrix metalloproteinase-9; mtDNA, mitochondrial DNA; NADPH, reduced nicotinamide adenine dinucleotide phosphate; NETs, neutrophil extracellular traps; NF-κB, nuclear factor kappa B; NK, natural killer; NLRP3, NOD-like receptor family pyrin domain-containing 3; NMDAR, N-methyl-D-aspartate receptor; NRF2, nuclear factor erythroid 2-related factor 2; O_2_•−, superoxide anion; OPC, oligodendrocyte precursor cell; OXPHOS, oxidative phosphorylation; PINK1, PTEN-induced kinase 1; PPAR, peroxisome proliferator-activated receptor; PPP, pentose phosphate pathway; ROS, reactive oxygen species; SDH, succinate dehydrogenase; STAIR, Stroke Therapy Academic Industry Roundtable; TCA, tricarboxylic acid cycle; TGF-β, transforming growth factor beta; TIMP-1, tissue inhibitor of metalloproteinases-1; TNF-α, tumor necrosis factor alpha; TREM2, triggering receptor expressed on myeloid cells 2; Treg, regulatory T cell; VWF, von Willebrand factor; ZO-1, zonula occludens-1; ΔΨm, mitochondrial membrane potential.

Neural plasticity and synaptic remodeling involve endogenous neural stem cells and glial–neuronal interactions. Experimental transcription-factor-mediated astrocyte-to-neuron reprogramming has generated neuron-like cells in rodent cortex and striatum and has been associated with functional improvement, but lineage fidelity, reproducibility, and clinical feasibility remain unresolved (45, 46). Pericytes and endothelial cells support microvessel formation through vascular endothelial growth factor (VEGF)- and microRNA (miRNA)-related mechanisms (47). Reactive astrocytes can contribute to scar organization and lesion containment, whereas other astrocyte states provide trophic and platelet-derived growth factor (PDGF)-related support for axonal and vascular remodeling; these functions vary with time, region, and transcriptional state (48). Chronic recovery therefore reflects coordinated neural plasticity, angiogenesis, white-matter repair, and scar remodeling, while persistent low-grade inflammation may remain in selected models and patient subsets (**Figure 1**).

## 3 Neurovascular-unit remodeling and microcirculatory failure

### 3.1 Neuronal injury and synaptic-network reorganization

After IS, neurons represent the major functional output unit of NVU injury. The extent of neuronal death and preservation of synaptic networks influence infarct expansion and recovery potential. Severe flow interruption and energy depletion rapidly render core neurons irreversibly injured, whereas penumbral neurons retain partial metabolic activity and structural plasticity and therefore remain a principal target for reperfusion-based tissue salvage (49).

During the hyperacute and acute phases, excitotoxicity, Ca^2+^ overload, and mitochondrial injury initially disrupt neuronal homeostasis and subsequently impair synaptic structure and function. Ischemic brain injury has been shown to induce neuronal mitochondrial damage and apoptotic responses, contributing to cognitive and motor dysfunction (50). In parallel, cerebral ischemia promotes the formation of cofilin–actin rods, which disturb the actin cytoskeleton and synaptic architecture, thereby reducing the efficiency of synaptic transmission (51). During excitotoxic stress, key synaptic proteins also undergo proteolytic cleavage and altered expression, affecting presynaptic neurotransmitter release, postsynaptic receptor localization, and overall network stability (52). These findings indicate that neuronal injury after IS is not limited to cell death; it also involves synaptic disconnection and local circuit disintegration.

Despite early disruption of synaptic structures, penumbral neurons retain a degree of remodeling capacity. Brain-derived neurotrophic factor (BDNF), growth-associated protein 43, and postsynaptic density protein 95 are closely associated with dendritic spine formation, axonal regeneration, and restoration of postsynaptic density, and are therefore widely used as markers of post-stroke neural plasticity.

Post-stroke neural network reconstruction also depends on restoration of the excitatory–inhibitory neurotransmission balance. γ-aminobutyric acid (GABA) signaling can suppress excessive excitation, stabilize neural circuits, and reduce the risk of neuronal death, thereby creating a permissive environment for synaptic reconnection in the penumbra (53). Synaptic-network repair therefore requires both neurotrophic support and re-establishment of the dynamic balance between excitatory and inhibitory transmission.

Structurally, dendrites, axons, and synaptic terminals of penumbral neurons are not completely lost after ischemia; instead, they remain in a plastic state in which injury and repair coexist. Metabolic status is fundamental to synaptic activity, as ATP availability, mitochondrial function, and local energy metabolism directly influence synaptic transmission and maintenance of plasticity (54). Neuronal injury and synaptic-network reorganization therefore constitute the biological basis of NVU functional recovery, while astrocytes, endothelial cells, pericytes, and the ECM further shape this process through metabolic support, barrier stabilization, and microenvironmental remodeling.

### 3.2 Metabolic-support and barrier-regulatory functions of astrocytes

Astrocytes are key cellular components of the NVU, linking neurons, vascular endothelial cells, pericytes, and the immune microenvironment. After IS, astrocytes rapidly respond to hypoxia, energy substrate deprivation, glutamate accumulation, and oxidative stress. Through metabolic support, neurotrophic factor release, antioxidant defense, and BBB regulation, they participate in both injury containment and tissue repair (55, 56). During the acute ischemic phase, astrocytic regulation of glutamate homeostasis and energy metabolism is particularly important. Evidence indicates that glutamate can serve as an alternative substrate to support ATP production in astrocytes under oxygen–glucose deprivation; however, sustained glutamate exposure may also induce reactive astrogliosis, underscoring the dual protective and injurious potential of astrocyte responses (57).

Astrocytes contribute to post-stroke neuroprotection through metabolic reprogramming. After ischemia–reperfusion, reactive astrocytes typically exhibit enhanced glycolysis, reduced glutamate-uptake capacity, and increased antioxidant burden. Their metabolic state directly influences neuronal survival, inflammatory responses, and BBB stability (58).

Beyond metabolic support, astrocyte-derived neurotrophic factors provide an essential basis for neuronal survival and network reconstruction after stroke. Reactive astrocytes can release glial cell line-derived neurotrophic factor (GDNF), thereby promoting neuronal survival after ischemic injury, reducing mitochondrial fission and caspase-dependent apoptosis, and improving motor recovery (59). Conversely, astrocyte-specific deletion of GDNF aggravates cerebral infarction, oxidative stress, and long-term functional impairment (60).

Astrocytic regulation of the BBB is bidirectional. Astrocytic endfeet support endothelial junctions through mediators such as angiopoietin-1, GDNF, IGF-1, and ApoE, whereas intense inflammatory or ischemia–reperfusion stress can induce VEGF, MMPs, nitric oxide, and endothelin-1 and thereby increase barrier permeability (61). Direct experimental work further showed that astrocyte-derived pentraxin 3 increased tight-junction protein expression and reduced endothelial permeability in a rat stroke model and astrocyte–endothelial coculture (62).

### 3.3 Endothelial injury and BBB disruption

After IS, brain microvascular endothelial cells (BMECs) become active regulators of BBB integrity and the NVU microenvironment. Oxygen–glucose deprivation and reperfusion impair mitochondrial and redox homeostasis, disrupt tight-junction organization, and increase leukocyte adhesion and transendothelial migration (63). Nuclear factor kappa B (NF-κB)- and JAK2/STAT3-associated inflammatory programs can increase cytokines and adhesion molecules, whereas PI3K/Akt-, BDNF/TrkB-Akt-, and nuclear factor erythroid 2-related factor 2 (NRF2)/HO-1-related signaling may support endothelial survival and antioxidant defense (63, 64). Endothelial P-glycoprotein-mediated suppression of autophagy has also been linked to barrier injury in experimental stroke (65). These observations represent model-specific mechanisms rather than a single clinically validated endothelial cascade.

Loss of tight-junction and basement-membrane integrity permits edema, leukocyte entry, and deterioration of the penumbral microenvironment. Distinct preclinical intervention studies have associated BBB preservation with inhibition of endothelial inflammatory signaling (64) or the caveolin-1/CD147/VEGFR2/MMP pathway (64, 66). Because these results were obtained with different interventions and models, they should be interpreted as pathway-specific preclinical observations rather than evidence that all components form one validated cascade. Systems-level analyses further support dynamic endothelial programs involving inflammation, survival, junctional control, and vascular remodeling (67).

### 3.4 Pericytes, capillary stalling, and microcirculatory no-reflow

Pericytes are specialized contractile cells located around capillaries and play essential roles in maintaining BBB integrity and regulating microcirculatory blood flow (68, 69). Under physiological conditions, pericytes support microvascular homeostasis and fine-tune local perfusion by closely ensheathing endothelial cells, secreting ECM components, and modulating capillary tone (69, 70). After IS, successful recanalization of large vessels may partially restore macroscopic blood flow; however, microcirculatory perfusion often remains insufficient, a condition commonly referred to as the “no-reflow” phenomenon (71, 72). This persistent perfusion deficit is closely linked to aberrant pericyte contraction and pericyte injury.

Recanalization of an occluded artery does not guarantee restoration of nutritive capillary flow. During ischemia and early reperfusion, oxidative and nitrosative stress can induce persistent pericyte contraction, endothelial and astrocytic end-foot swelling can narrow the capillary lumen, and platelet-rich microthrombi can impair downstream perfusion. In parallel, activated neutrophils adhere to the post-capillary and capillary endothelium and may physically obstruct capillaries. Neutrophil depletion or adhesion blockade improved reperfusion after recanalization, identifying capillary stalling as a no-reflow mechanism (73). These mechanisms are not mutually exclusive: pericyte constriction, endothelial swelling, platelet–leukocyte aggregates, NETs, and neutrophil plugging can converge to maintain tissue hypoxia despite macrovascular patency.

Pericyte dysfunction also reshapes the perivascular microenvironment. Multiple signaling pathways regulate pericyte morphology and contractile behavior. For example, upregulation of phosphoinositide 3-kinase δ (PI3Kδ) activates the Ca^2+^ channel transient receptor potential vanilloid 2 (TRPV2) through a tumor necrosis factor-α (TNF-α)-dependent mechanism, thereby enhancing pericyte contraction and worsening microcirculatory hypoperfusion (74). In parallel, integrin α8 and PDGFB/PDGFR-β signaling are critical for regulating pericyte morphology and vascular coverage, both of which are required for maintaining BBB integrity and microcirculatory function (70, 75). These findings indicate that pericytes are not only regulators of vascular blood flow but also central contributors to microvascular homeostasis and NVU repair.

Importantly, microcirculatory perfusion failure is not confined to the acute phase; it may persist into the subacute and chronic phases. Delayed pericyte loss is independently associated with reduced capillary perfusion, suggesting that pericyte death and microvascular dysfunction may be temporally dissociated (71). Accordingly, therapeutic strategies such as rapamycin and iptakalim have shown potential to restore microcirculatory perfusion by suppressing pericyte contraction, improving blood flow, and preserving BBB integrity (76, 77). These studies highlight that, even after successful large-vessel recanalization, pericyte function and microcirculatory status remain key determinants of stroke outcome.

In addition, interactions among pericytes, endothelial cells, and glial cells further shape microcirculatory homeostasis. Interventions such as EC-Exo and McM/RNPs enhance pericyte–endothelial communication by activating signaling pathways including PDGF–PDGFRβ and angiopoietin-1/angiopoietin-2–Tie2 (Ang1/Ang2–Tie2), thereby promoting capillary flow restoration and BBB repair (78, 79). In clinical contexts, protecting pericytes and modulating their contractile and reparative functions may represent an important therapeutic direction for improving microcirculatory perfusion, reducing ischemia–reperfusion injury, and enhancing neurological recovery after IS.

### 3.5 Extracellular-matrix and perivascular remodeling

After IS, the NVU ECM undergoes marked remodeling. The vascular basement membrane contains collagen IV, laminin, fibronectin, and other components required for endothelial stability and barrier organization (80, 81). Ischemia–reperfusion activates MMP-2 and MMP-9, promoting ECM degradation, basement-membrane disruption, and increased BBB permeability (82, 83). MMP-9-mediated degradation of ECM and junctional proteins is associated with edema, neurological deterioration, and hemorrhagic transformation (83).

ECM remodeling contributes both to acute barrier failure and to later tissue repair. Neutrophils and monocytes/macrophages can release MMP-9. Early human transcriptomic integration and quantitative polymerase chain reaction validation showed co-enrichment of MMP-9 with oxidative-stress and neutrophil-response genes (84), providing associative rather than causal evidence. In experimental thrombolysis-associated BBB injury, MMP-9-mediated degradation of collagen IV and laminin contributes to microvascular leakage and hemorrhagic transformation (82, 84). Inhibiting MMP-9 or restoring ECM homeostasis may be protective, but benefit depends on treatment timing and reperfusion status.

**Figure 2:**
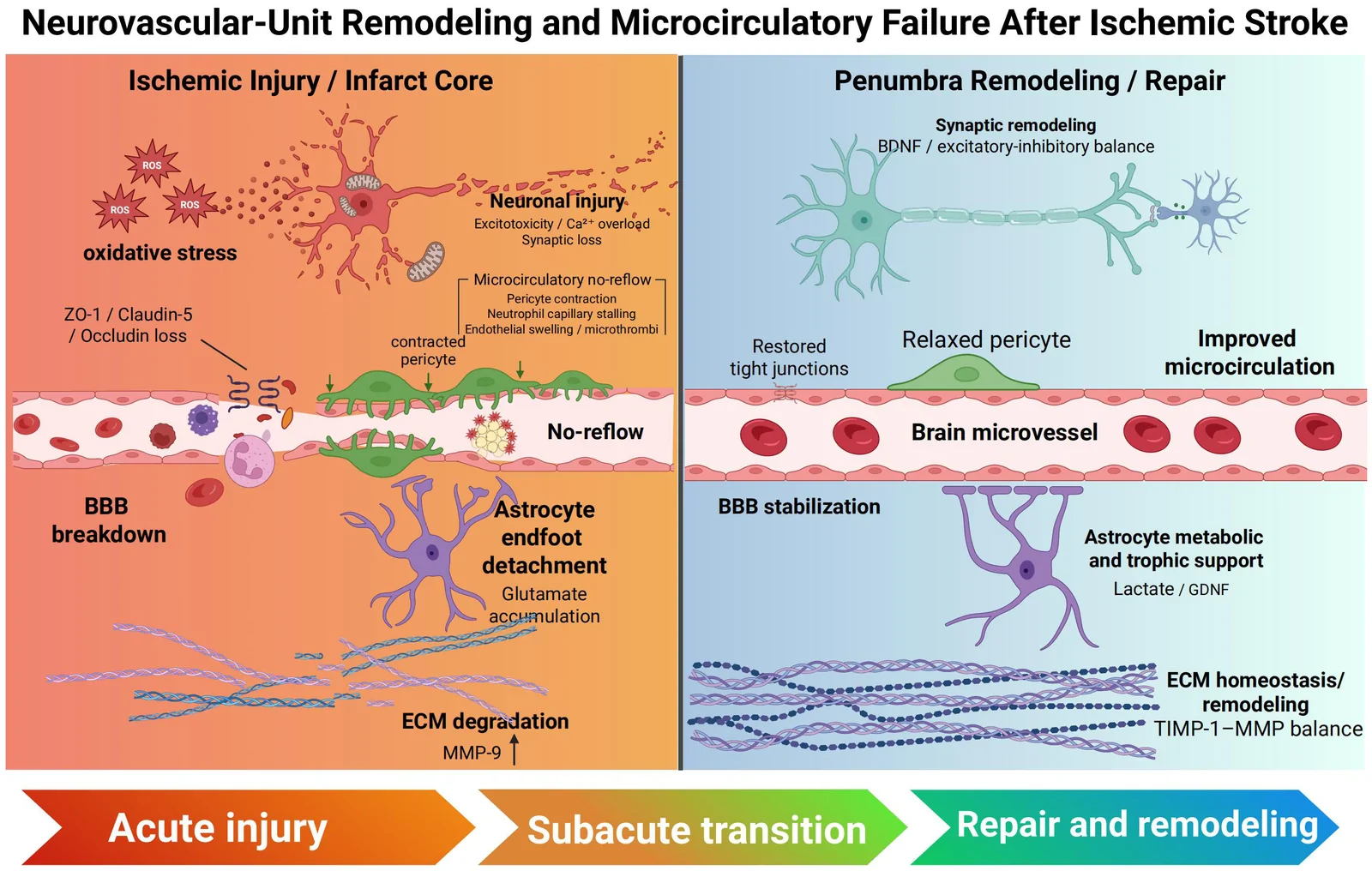
Neurovascular-unit remodeling and microcirculatory failure after ischemic stroke. The orange left half represents acute ischemic injury in the infarct core, whereas the blue-to-pale-green right half represents penumbral remodeling and repair. The black central divider separates these conceptual fields, and the large colored arrows along the bottom indicate progression from acute injury through subacute transition to repair and remodeling. In the injured core, ROS-associated oxidative stress, excitotoxicity, and Ca^2+^ overload accompany neuronal injury and synaptic loss. Loss of ZO-1, claudin-5, and occludin illustrates BBB breakdown; contracted pericytes, neutrophil capillary stalling, endothelial swelling, and microthrombi converge on microcirculatory no-reflow. Astrocyte endfoot detachment and glutamate accumulation are shown together with MMP-9 upregulation and ECM degradation. In the penumbral repair field, restored tight junctions and relaxed pericytes support BBB stabilization and improved microcirculation. Synaptic remodeling is associated with BDNF and restoration of excitatory-inhibitory balance; astrocytes provide lactate- and GDNF-related metabolic and trophic support; and TIMP-1–MMP balance accompanies ECM homeostasis and remodeling. Pericytes are colored green in both fields to denote cell identity; contracted and relaxed states are conveyed by morphology and labels rather than by a color change. Local arrowheads indicate the directional relationships depicted within each field, whereas the large bottom arrows denote temporal progression. ACOD1/IRG1, aconitate decarboxylase 1/immune-responsive gene 1; ATP, adenosine triphosphate; BBB, blood–brain barrier; BDNF, brain-derived neurotrophic factor; Ca^2+^, calcium ion; cfDNA, cell-free DNA; cGAS–STING, cyclic GMP–AMP synthase–stimulator of interferon genes; CH_3_, methyl group; CH25H, cholesterol 25-hydroxylase; Cu^2+^, copper(II) ion; DAMPs, damage-associated molecular patterns; dsDNA, double-stranded DNA; ECM, extracellular matrix; EVs, extracellular vesicles; Fe^2+^, ferrous ion; G6PD, glucose-6-phosphate dehydrogenase; GABA, γ-aminobutyric acid; GDNF, glial cell line-derived neurotrophic factor; GPX4, glutathione peroxidase 4; GSH, glutathione; HT, hemorrhagic transformation; IL, interleukin; IV, intravenous; LNPs, lipid nanoparticles; M1/M2, conventional labels for classically/alternatively activated myeloid states; miRNAs, microRNAs; MMP, matrix metalloproteinase; MMP-9, matrix metalloproteinase-9; mtDNA, mitochondrial DNA; NADPH, reduced nicotinamide adenine dinucleotide phosphate; NETs, neutrophil extracellular traps; NF-κB, nuclear factor kappa B; NK, natural killer; NLRP3, NOD-like receptor family pyrin domain-containing 3; NMDAR, N-methyl-D-aspartate receptor; NRF2, nuclear factor erythroid 2-related factor 2; O_2_•−, superoxide anion; OPC, oligodendrocyte precursor cell; OXPHOS, oxidative phosphorylation; PINK1, PTEN-induced kinase 1; PPAR, peroxisome proliferator-activated receptor; PPP, pentose phosphate pathway; ROS, reactive oxygen species; SDH, succinate dehydrogenase; STAIR, Stroke Therapy Academic Industry Roundtable; TCA, tricarboxylic acid cycle; TGF-β, transforming growth factor beta; TIMP-1, tissue inhibitor of metalloproteinases-1; TNF-α, tumor necrosis factor alpha; TREM2, triggering receptor expressed on myeloid cells 2; Treg, regulatory T cell; VWF, von Willebrand factor; ZO-1, zonula occludens-1; ΔΨm, mitochondrial membrane potential.

Beyond MMP-mediated degradation, the ECM contributes to a reparative perivascular environment. TIMP-1 can regulate ECM homeostasis, endothelial stability, astrocyte responses, and neuroregeneration, but its effects depend on pathological context and timing: restraining excessive proteolysis may be beneficial acutely, whereas persistent elevation may accompany fibrosis or scar formation later (85). The IL-4-conditioned-macrophage mechanism underlying early fibrotic-scar formation is described in *Section 2.3*; here, its ECM implication is emphasized: excessive matrix deposition may later limit axonal regeneration and long-term recovery (**Figure 2**).

## 4 Inflammation-centered immune remodeling across brain and peripheral compartments

Post-ischemic inflammation is generated by coordinated responses in the brain, blood, bone marrow, spleen, gut, and autonomic–endocrine systems. Within the brain, resident microglia, astrocytes, endothelial cells, pericytes, oligodendroglial-lineage cells, and neurons all participate in inflammatory sensing and signaling. In parallel, circulating neutrophils, monocytes, lymphocytes, and platelets interact with activated endothelium and enter the ischemic territory in a time- and compartment-dependent manner. The biological effects of these cells cannot be classified as uniformly detrimental or beneficial, because their functions depend on activation state, anatomical location, stroke stage, reperfusion status, age, sex, and metabolic comorbidity.

### 4.1 Microglia and infiltrating MDMs

After IS, microglia, as resident innate immune cells of the brain, are among the earliest cellular populations to sense ischemic injury and local microenvironmental perturbations. Ischemia and reperfusion stimuli drive microglia from a homeostatic surveillant state toward reactive phenotypes, enabling the release of inflammatory cytokines and chemokines that amplify local inflammation. At the same time, microglia contribute to tissue recovery by phagocytosing necrotic cells, myelin debris, synaptic remnants, and infiltrating neutrophils, thereby facilitating debris clearance and inflammatory resolution (41, 86). Thus, microglia should not be viewed simply as proinflammatory cells; rather, they exert stage-dependent functions in injury amplification, debris removal, and reparative regulation throughout the post-stroke course.

Recent single-cell sequencing and fate-mapping studies have shown that microglial activation can no longer be adequately captured by the traditional M1/M2 dichotomy. After ischemic injury, microglia diversify into multiple subpopulations with distinct transcriptional signatures, proliferative capacities, and functional orientations. These states are shaped jointly by hypoxia, cellular debris, lipid burden, inflammatory mediators, and intercellular communication (87). Multicolor fate-mapping studies further reveal polyclonal microglial expansion after ischemia, with different clones displaying divergent electrophysiological properties and cell–cell interaction patterns during recovery. These findings suggest that microglial functional heterogeneity arises not only from phenotypic transitions but also from local clonal expansion and spatial organization (88).

Phagocytic clearance is a central mechanism through which microglia contribute to repair. Triggering receptor expressed on myeloid cells 2 (TREM2) signaling supports recognition of lipid-rich debris, phagocytosis, inflammatory regulation, and cell survival (89). In experimental ischemic stroke, TREM2–IGF-1 signaling enhanced microglial glucose metabolism and neuroprotective function (90). By contrast, TREML2 can produce different effects: Treml2 deficiency alleviated demyelination and cognitive impairment during chronic cerebral hypoperfusion (91). ChemR23/p38 MAPK, P2X4R, and L-PGDS–PGD2–DP1 signaling also regulate microglial/macrophage phagocytosis, underscoring the dependence of debris clearance on receptor, ligand, and tissue context (92, 93).

Microglial functional states are further shaped by epigenetic regulation, the autophagy–lysosomal system, and bioenergetic supply. Histone deacetylase 1 imbalance promotes proinflammatory microglial activation and aggravates post-stroke neuroinflammation, whereas HDAC3 deficiency enhances chemotactic signaling, recruits macrophages, accelerates myelin-debris clearance, and promotes white-matter repair (94, 95). In parallel, NLRC5 sustains proinflammatory microglial responses by impairing lysosomal function and autophagic flux (96). These findings indicate that inflammatory and reparative microglial functions are jointly determined by phagocytic receptors, intracellular degradation systems, and cellular energy state.

Monocytes subsequently infiltrate the brain and differentiate into macrophages, with functions that depend on time and tissue context. Early after stroke they can amplify local inflammation; during subsequent repair they participate in clearance of necrotic cells and myelin debris and may release proangiogenic factors (97). Aging is another determinant of peripheral immune responses. In a distal-MCAO study comparing young and aged male mice, monocytes from aged mice showed lower oxidized nicotinamide adenine dinucleotide (NAD+) availability and greater inflammatory responses. Four weeks of prestroke nicotinamide-riboside supplementation restored monocyte NAD+ and reduced systemic and brain inflammation in aged mice. These findings support an age-dependent experimental mechanism but do not establish efficacy in patients or after post-onset treatment (98).

### 4.2 Astrocytes, oligodendrocytes, and OPCs as immune effectors

Astrocytes are active immune effectors rather than passive components of the NVU. Ischemia-induced ATP release, cytokines, complement signals, and DAMPs activate astrocytic NF-κB, JAK/STAT, and inflammasome-associated pathways. Reactive astrocytes can secrete IL-6, CXCL1, CCL2, complement C3, MMPs, and vascular permeability-regulating factors, thereby influencing leukocyte recruitment, microglial activation, endothelial integrity, and neuronal survival. Activated microglia can induce a neurotoxic astrocyte program through IL-1α, TNF, and C1q signaling (99), whereas other astrocyte states support glutamate clearance, antioxidant defense, lactate delivery, scar organization, and tissue repair. In experimental stroke, microglia-derived IL-1 receptor antagonist suppressed astrocytic CXCL1 production, reduced neutrophil recruitment and microvascular obstruction, and improved reperfusion (100). The binary A1/A2 terminology should therefore be used cautiously; transcriptomic state, timing, and functional output are more informative than a fixed harmful-versus-protective label.

Oligodendroglial-lineage cells also participate in inflammatory and metabolic remodeling. Mature oligodendrocytes are highly vulnerable to energy failure, oxidative stress, excitotoxicity, and inflammatory mediators, and their loss contributes to axonal conduction failure and delayed white-matter degeneration (101). OPCs proliferate and migrate toward ischemic white matter, but only a subset differentiates into remyelinating oligodendrocytes, indicating that inflammatory and vascular cues constrain effective repair (102). OPCs can interact with endothelial cells and influence BBB integrity; experimentally transplanted OPCs reduced barrier disruption after cerebral ischemia (103). In the reparative phase, CD11c-positive microglia and regulatory T cell-derived osteopontin have been linked to oligodendrogenesis and white-matter recovery (36, 104). These observations position the oligodendroglial lineage at the intersection of inflammation, axonal metabolic support, barrier remodeling, and chronic functional recovery.

### 4.3 Neutrophils, monocytes, and platelet–leukocyte interactions

Neutrophils contribute to ischemic injury through mechanisms that extend beyond NETs. Following endothelial activation, neutrophils undergo selectin-dependent rolling, integrin-mediated adhesion, platelet–neutrophil aggregation, degranulation, and release of ROS, elastase, myeloperoxidase, and MMPs. These processes can damage the endothelial glycocalyx and basement membrane, increase BBB permeability, and obstruct capillary flow. In experimental stroke, capillary-obstructing neutrophils contribute to incomplete microvascular reperfusion despite reopening of the upstream artery (73). NET formation further promotes immunothrombosis and can impair vascular remodeling (29, 30), but NETosis represents one component of a broader neutrophil effector program.

Monocyte recruitment generally follows the early neutrophil-dominated response. Circulating monocytes adhere to activated endothelium, enter the ischemic territory, and differentiate into macrophages whose inflammatory, phagocytic, and reparative functions depend on time, tissue compartment, and local metabolic conditions. Their recruitment kinetics and vascular interactions should therefore be distinguished from the transcriptional states of resident microglia and established MDMs described in *Section 4.1*.

### 4.4 Adaptive lymphocytes: T cells, B cells, and natural killer cells

T lymphocytes display temporally and functionally heterogeneous responses after IS. During the acute phase, conventional CD4+ and CD8+ T cells can promote endothelial activation, microvascular dysfunction, and tissue injury through cytokine-dependent and, in some models, antigen-independent mechanisms (105). Persistent activation and delayed CD8+ T-cell entry may contribute to chronic neuroinflammation (106, 107). By contrast, Tregs can limit excessive inflammation and later support control of astrocyte reactivity, white-matter repair, and tissue remodeling (108). In mouse tMCAO, microglial LILRB4 limited CCL2-dependent CD8+ T-cell recruitment and activation (109). Therapeutic effects therefore depend on lymphocyte subset, compartment, and phase.

B-cell effects are similarly context-dependent. IL-10-producing regulatory B cells reduced inflammatory injury in experimental stroke (110), whereas a separate study using pharmacological depletion, B-cell-deficient mice, and adoptive transfer found no major effect of B cells on infarct volume during the first 1–3 days (111). In contrast, delayed B-cell accumulation and antibody-producing responses have been associated with post-stroke cognitive impairment (112). B cells may therefore be dispensable for early infarct expansion in some models yet relevant to immune regulation, chronic neuroinflammation, and cognitive sequelae.

NK cells bridge innate and lymphoid immunity. NK cells may exacerbate acute ischemic injury through cytotoxic mediators and inflammatory interactions with neurons and endothelial cells (113). However, an endothelial CXCL12-dependent protective NK-cell response has also been reported, indicating that vascular localization and activation state can alter function (114). Human observations have linked CXCL8/CXCR2 signaling to granulocyte and NK-cell recruitment after IS (115). NK cells should therefore be regarded as heterogeneous regulators rather than uniformly injurious effectors.

### 4.5 Soluble inflammatory mediators and innate immune sensors

After IS, inflammatory mediators and innate sensors link cellular injury, metabolic disturbance, and immune activation. DAMPs released by injured neural and vascular cells can engage TLR4/NF-κB signaling and induce IL-1β, IL-6, and TNF-α, thereby initiating local neuroinflammation (116). The magnitude and consequence of this signaling depend on DAMP identity, cellular receptor expression, compartment, and disease phase.

Within this inflammatory cascade, NF-κB functions not only as a core regulator of early proinflammatory transcription but also as an important upstream signal for NLRP3 inflammasome activation. Once activated, the NLRP3 inflammasome promotes caspase-1 cleavage and IL-1β release, and further amplifies ischemia–reperfusion injury through gasdermin D (GSDMD)-mediated pyroptosis (117). Consistently, inhibition of the NF-κB/NLRP3 axis reduces neuronal pyroptosis, decreases infarct volume, and improves neurological function, supporting this axis as a key mechanism of acute inflammatory amplification (117). In addition, gut-derived toxins, oxidative stress, and intracellular damage signals can influence vascular barrier integrity and the immune microenvironment through NLRP3-dependent pathways. These findings suggest that inflammasome activation is not merely an intracellular event, but rather a shared signaling node that connects the brain–gut axis, vascular dysfunction, and immune responses (118).

Beyond TLR- and inflammasome-associated pathways, cyclic GMP–AMP synthase–stimulator of interferon genes (cGAS–STING) signaling links mitochondrial injury and cytosolic DNA sensing to post-stroke immune activation. Genetic and mechanistic studies indicate that cGAS–STING activation can amplify microglial inflammatory signaling and autophagy-related dysfunction after experimental ischemia, whereas disruption of pathway components attenuates inflammatory injury (119, 120). These findings support a causal preclinical pathway but do not establish the efficacy of any specific compound in patients.

Circulating cell-free DNA (cfDNA) extends the DAMP framework beyond intracellular DNA sensing. cfDNA can arise from necrotic or apoptotic cells, active secretion, and NET formation, and may engage TLR9, cGAS–STING, or absent in melanoma 2 (AIM2) pathways according to its source, localization, and molecular packaging. In a small human acute-IS cohort, plasma cfDNA was higher than in stroke mimics and correlated with infarct volume and neutrophil count (121). In 166 thrombectomy-treated patients, cfDNA, DNase activity, MPO–histone complexes, and thrombus NET burden showed selected associations, but circulating cfDNA was not a thrombus-specific or NET-specific readout (122). cfDNA should therefore be interpreted as a source-heterogeneous injury and inflammatory biomarker, not as proof of a single DNA-sensing pathway.

miRNAs provide a second layer of immune regulation by coordinating multiple targets within and between neurons, glia, vascular cells, and leukocytes. Human serum miR-124, miR-9, and miR-219 levels have been associated with infarct volume, high-sensitivity C-reactive protein, and MMP-9, although these observations are associative (123). In mouse stroke models, microglial small extracellular vesicle miR-124 regulated astrocytic STAT3 and glial-scar formation (124), while astrocyte-derived exosomal miR-378a-5p modulated NLRP3-related neuronal pyroptosis (125). These findings illustrate network regulation but do not justify treating a circulating miRNA concentration as a cell-specific causal mechanism without source tracing and target validation.

Inflammatory cytokines themselves also exert both amplifying and regulatory effects. Interleukin-17A (IL-17A) promotes aberrant angiogenesis and inflammatory cell infiltration during the acute phase, and aggravates vascular hyperpermeability and tissue injury by upregulating MMP-9 (126). TNF-α is a shared ligand for tumor necrosis factor receptor 1 (TNFR1) and tumor necrosis factor receptor 2 (TNFR2), yet these receptors mediate markedly different biological effects. Excessive activation of TNF-α/TNFR1 signaling during the acute phase primarily promotes inflammation, oxidative stress, and cell death, whereas TNF-α/TNFR2 signaling is more closely associated with immune regulation, vascular repair, and tissue regeneration. Thus, the role of TNF-α after stroke is strongly time-window- and receptor-dependent (127). In addition, complement and chemokine networks participate in immune-cell recruitment and inflammatory propagation. For example, complement component 5a receptor 1 (C5aR1) and the interferon regulatory factor 7/C-X-C motif chemokine ligand 10 (IRF7–CXCL10) axis have been closely linked to microglial inflammatory states and immune-cell migration (128, 129).

### 4.6 Stroke-induced immunodepression and peripheral-organ responses

Cerebral ischemia elicits a biphasic systemic immune response in which early inflammatory activation can be followed by profound immunodepression. Brain injury activates sympathetic outflow and the hypothalamic–pituitary–adrenal axis, increasing catecholamine and glucocorticoid signaling. In experimental stroke, sympathetic activation drives lymphocyte apoptosis and functional immune suppression, thereby increasing susceptibility to bacterial infection (130). This neurogenic immune-deficiency syndrome includes lymphopenia, monocyte deactivation, altered antigen presentation, splenic contraction or cellular redistribution, and impaired antibacterial defense (131).

The systemic response is clinically important because pneumonia and urinary-tract infection can worsen neurological outcome and complicate interpretation of inflammatory biomarkers. Human studies indicate that immunodepression and infection are intertwined with stroke severity and functional outcome (132). Experimental evidence further suggests that disruption of intestinal barriers and translocation of commensal bacteria can contribute to post-stroke infection (133). Systemic inflammation and systemic immunosuppression are therefore not mutually exclusive and may occur in different compartments or phases of the same patient.

Baseline host factors shape the magnitude and phenotype of post-stroke inflammation. Aging, sex, smoking, obesity, diabetes, dyslipidemia, hypertension, and chronic infection or inflammatory disease can alter endothelial activation, leukocyte priming, immunometabolic reserve, and the balance between tissue injury and repair. These factors are distributed differently between experimental cohorts and clinical populations and should be treated as biological modifiers of the neuroimmune response rather than only as epidemiological stroke risks.

### 4.7 Gut–brain immune communication

The gut–brain axis provides a bidirectional route through which stroke alters systemic immunity and peripheral metabolism. Cerebral ischemia can disrupt autonomic regulation, intestinal motility, epithelial barrier integrity, and microbial community structure. In turn, microbial products and metabolites influence intestinal lymphocyte differentiation, circulating cytokines, and leukocyte trafficking to the ischemic brain. Experimental studies have shown that microbiota-dependent γδ T-cell and IL-17 responses can modify ischemic injury (134) and that stroke-induced dysbiosis can amplify neuroinflammation (135). Conversely, a microbiota configuration favoring regulatory immune responses can be protective in experimental models (136).

Gut-derived signals should be integrated with systemic immunodepression and metabolic remodeling. Stroke alters intestinal immune-cell trafficking (137), while barrier failure may permit bacterial translocation and contribute to post-stroke infection (133). Short-chain fatty acids, bile-acid signaling, tryptophan metabolites, and endotoxin exposure are plausible mediators, but causal evidence in patients remains limited. Microbiome modulation should therefore be described as an emerging translational direction rather than a clinically established intervention.

### 4.8 Human–rodent differences and human intracranial evidence

Human–rodent concordance and divergence require explicit consideration. A systematic review and meta-analysis comparing 20 human studies with 188 mouse and rat studies found partial conservation of the post-ischemic leukocyte sequence: macrophage/monocyte and neutrophil accumulation generally preceded lymphocytic influx, and these myeloid populations predominated during the first 72 h. However, both human and rodent data were highly heterogeneous, and leukocyte density in experimental stroke varied with species, occlusion site, reperfusion, and infarct volume (138). Differences in age, comorbidity, immune-system composition, lesion heterogeneity, microbiota, supportive care, and sampling time further constrain direct translation.

Mechanical thrombectomy has created a unique opportunity to interrogate the human neurovascular–immune environment during the hyperacute phase. Registries such as Blood and Clot Thrombectomy Registry and Collaboration collect intracranial blood and thrombotic material from the occluded vascular territory together with systemic arterial samples (139). Feasibility studies have identified leukocyte populations in thrombectomy-derived intracranial blood (140), local platelet activation and chemokine release (141), cerebral leukocyte populations (142), and intracranial enrichment of vascular and inflammatory markers such as vascular cell adhesion molecule 1 (143).

These datasets narrow the gap between rodent mechanisms and human biology, but thrombectomy cohorts predominantly represent large-vessel occlusion, samples are obtained after variable delays and treatment exposures, and intracranial blood does not reproduce the cellular composition of infarcted parenchyma. Nevertheless, paired intracranial–systemic sampling provides a major translational platform for evaluating local thromboinflammation.

Recent human tissue and thrombus studies extend this platform but also define its limits. Single-nucleus RNA sequencing of peri-infarct cortex obtained during decompressive craniectomy within approximately 24 h identified region-specific astrocyte, oligodendrocyte, and oligodendrocyte-precursor responses (144). Because that study included only two stroke samples and used trauma tissue as the comparator, it provides a valuable feasibility map rather than a population-level atlas. In parallel, spatial transcriptomic analysis of thrombectomy-retrieved thrombi linked large-artery-atherosclerotic thrombi to a profibrotic macrophage program and cardioembolic thrombi to greater neutrophil activation and NET-associated signaling (145). These findings support etiologic stratification, while emphasizing that thrombus biology, intracranial blood, peri-infarct tissue, and systemic blood sample different but complementary compartments.

### 4.9 Etiology- and subtype-dependent remodeling

IS not a single immunobiological entity. Large-artery atherosclerotic, cardioembolic, small-vessel, cancer-associated, and other strokes begin with different vascular substrates and can differ in clot architecture, endothelial activation, systemic inflammatory context, collateral status, and response to reperfusion. In a histological analysis of 501 thrombectomy-retrieved thromboemboli, cardioembolic clots contained fewer red blood cells but more platelets, fibrin, leukocytes, and extracellular DNA than large-artery atherosclerotic clots; nevertheless, individual components did not reliably identify etiology (146). Paired thrombus–plasma lipidomics likewise distinguished selected large-artery-atherosclerotic and cancer-associated clots from cardioembolic clots through local thrombus lipids, whereas corresponding plasma differences were limited (147).

Etiologic heterogeneity has two consequences for an integrative review. First, a mechanism observed in thrombectomy-treated large-vessel occlusion cannot automatically be generalized to lacunar stroke or to patients without reperfusion. Second, thrombus, intracranial blood, systemic blood, and parenchymal tissue capture different compartments and should not be treated as interchangeable biomarkers. Future mechanistic studies and trials should prespecify etiology, occlusion site, cancer or infection status, collateral grade, thrombolysis, thrombectomy, and reperfusion quality, while recognizing that current subtype-associated NET and multi-omics signatures remain hypothesis-generating (148).

### 4.10 Brain-border immunity and bidirectional meningeal lymphatic communication

Post-stroke immunity is not restricted to the blood and brain parenchyma. The meninges, skull marrow, cerebrospinal fluid, and cervical lymph nodes form an anatomically connected brain-border system. Skull and vertebral marrow can supply monocytes and neutrophils to the meninges and CNS through channels that are distinct from the systemic circulation (149), while cerebrospinal fluid can enter skull-marrow niches through dural channels and convey CNS-derived signals (150). After experimental ischemia–reperfusion, ipsilateral skull marrow shows regionally biased myeloid activation that is partly regulated by osteopontin signaling (151). Thus, the skull–meninges interface should be considered a local immune reservoir and sensing organ, not simply a passive barrier.

Causal stroke studies further indicate that brain-border pathways can regulate both recruitment and immune phenotype. In mice, a meningeal mast-cell receptor promoted degranulation, skull-marrow neutrophil recruitment, and subsequent migration from dura to brain; its human ortholog was detected in human meningeal mast cells, although therapeutic causality was established only in the animal model (152). A separate study combined experimental stroke with human skull-marrow sampling and implicated nociceptive neuron-derived calcitonin gene-related peptide in mobilizing myeloid-derived suppressor cells (153). Single-cell analyses also show that dural and brain-infiltrating IL-17-producing γδ T cells share lineage features but differ in migratory, proliferative, and effector states after stroke (154). These observations add a spatial dimension to inflammation: immune-cell origin and route of entry may influence function after the cells reach the injured brain.

Meningeal lymphatics provide a parallel efflux route for fluid, antigens, and immune cells, but their net effect is stage-dependent. Prophylactic vascular endothelial growth factor C (VEGF-C) expression enhanced drainage and improved outcome in mice, whereas acute post-stroke VEGF-C administration did not reproduce the benefit (155). Other models indicate that the endogenous VEGF-C–Flt4 response can contribute to acute swelling (156), that lymphatic disruption can reduce reperfusion-associated inflammation through altered CCN1 signaling (157), and that enhancing CCL2–CCR2-dependent macrophage clearance can improve edema and recovery (158). These apparently divergent findings argue against treating “more drainage” or “less drainage” as universally beneficial. The relevant variables include treatment timing, the transported cargo, lymph-node signaling, edema, and whether the dominant process is harmful immune recruitment or beneficial clearance.

### 4.11 Aging, sex, and immune reserve

Age and sex modify the baseline immune and metabolic state on which stroke acts. In single-cell studies, aged mouse microglia showed greater induction of the interferon-linked regulator Ifi27l2a after stroke; partial genetic deletion reduced gliosis, tissue injury, and functional deficits, and age- and stroke-associated expression was also examined in human autopsy tissue (159). A separate study in young male and female mice found substantial post-stroke microglial heterogeneity but no major global sex dimorphism, cautioning against assuming that every microglial state is sex-specific (160). Sex effects may instead emerge at selected pathways or recovery stages.

This pathway specificity is illustrated by microglial TNFR2 signaling, whose deletion had different effects on phagocytosis, myelin-debris clearance, oligodendrocyte maturation, and recovery in female and male mice; corresponding cerebrospinal-fluid associations were stronger in women in the studied patient cohort (161). Human blood transcriptomic data likewise show time-dependent, sex-related differences in X-chromosome gene expression during the first 24 h after IS (162). These studies do not justify separate therapeutic rules by sex alone, but they support prespecified sex-stratified analyses and the use of aged, comorbid models when validating immunomodulatory targets.

### 4.12 Temporal transition from injury amplification to immune resolution

Immune responses after IS exhibit pronounced time-window dependence. The same immune-cell population or signaling pathway may exert markedly different effects at distinct stages of disease progression. During the hyperacute and acute phases, injured neurons, glial cells, and vascular cells release DAMPs, which rapidly activate brain-resident microglia and promote the recruitment of peripheral neutrophils and monocytes to ischemic regions. Immune responses during this stage are characterized by inflammatory cytokine release, chemokine upregulation, NET formation, and local thromboinflammation, all of which can aggravate neuroinflammation and microenvironmental imbalance (8, 163). Notably, neutrophil-derived NETs not only contribute to acute thromboinflammatory responses but may also influence inflammatory features and prognostic assessment across different stroke subtypes (148).

As IS progresses into the subacute phase, immune responses gradually shift from inflammatory amplification toward debris clearance and the initiation of tissue repair. MDMs increase markedly during this stage and display strong phagocytic activity, accompanied by enriched expression of lysosome-associated genes. These properties enable MDMs to participate in the clearance of necrotic cells, myelin debris, and lipid-rich residues (32). Single-cell transcriptomic and chromatin accessibility analyses further indicate that subacute macrophages do not represent a uniform proinflammatory population; instead, they comprise multiple subsets with distinct clearance capacities and functional states (32). The expansion of repair-associated macrophages also suggests that appropriately regulated immune-cell infiltration may facilitate inflammatory resolution and tissue reconstruction (164).

**Figure 3:**
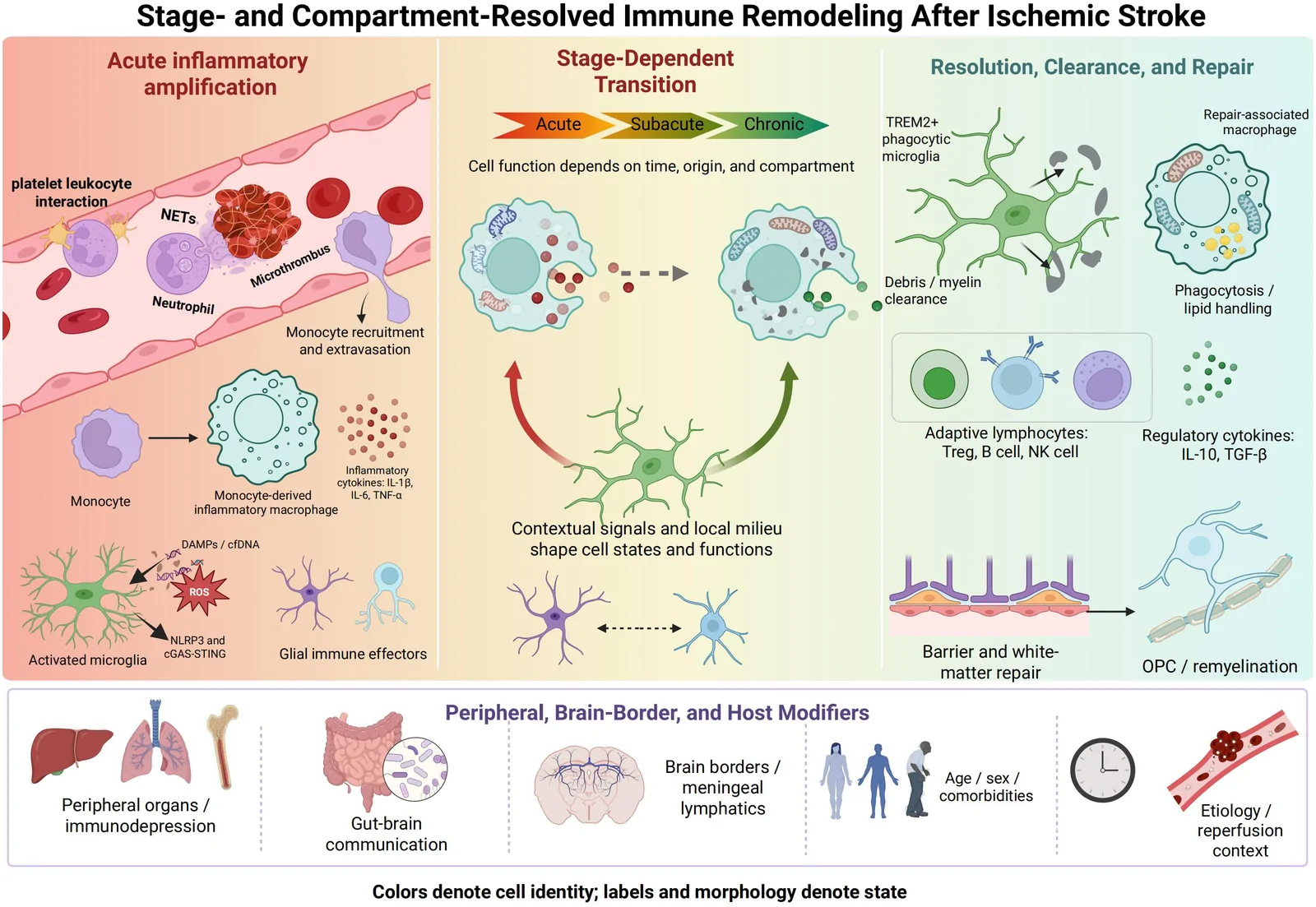
Stage- and compartment-resolved immune remodeling after ischemic stroke. The left compartment depicts acute inflammatory amplification. Platelet–leukocyte interactions, neutrophils, neutrophil extracellular traps, and microthrombus formation are shown within the vessel, together with monocyte recruitment and extravasation. In the parenchymal compartment, recruited monocytes give rise to monocyte-derived inflammatory macrophages associated with the production of IL-1β, IL-6, and TNF-α, while activated microglia and other glial immune effectors are linked to DAMP/cfDNA release, ROS, NLRP3, and cGAS–STING signaling. The middle compartment emphasizes a stage-dependent transition across the acute, subacute, and chronic phases: immune-cell function varies with time, cellular origin, anatomical compartment, contextual signals, and the local milieu. The illustrated myeloid-cell states and bidirectional glial communication therefore represent context-dependent functional transitions rather than fixed phenotypes. The right compartment summarizes resolution, clearance, and repair. TREM2-positive phagocytic microglia remove cellular and myelin debris; repair-associated macrophages participate in phagocytosis and lipid handling; Tregs, B cells, and NK cells contribute to adaptive immune regulation; IL-10 and TGF-β represent regulatory cytokines; and barrier and white-matter repair are linked to OPC-mediated remyelination. The lower band identifies peripheral, brain-border, and host modifiers, including peripheral-organ responses and immunodepression, gut–brain communication, brain borders and meningeal lymphatics, age, sex, comorbidities, stroke etiology, and the reperfusion context. Solid arrows indicate the direction of recruitment, clearance, transition, or functional linkage as depicted; dashed arrows indicate context-dependent state transition or bidirectional communication. Colors denote cell identity, whereas labels and morphology denote functional state. The plus sign denotes marker-positive cells. ACOD1/IRG1, aconitate decarboxylase 1/immune-responsive gene 1; ATP, adenosine triphosphate; BBB, blood–brain barrier; BDNF, brain-derived neurotrophic factor; Ca^2+^, calcium ion; cfDNA, cell-free DNA; cGAS–STING, cyclic GMP–AMP synthase–stimulator of interferon genes; CH_3_, methyl group; CH25H, cholesterol 25-hydroxylase; Cu^2+^, copper(II) ion; DAMPs, damage-associated molecular patterns; dsDNA, double-stranded DNA; ECM, extracellular matrix; EVs, extracellular vesicles; Fe^2+^, ferrous ion; G6PD, glucose-6-phosphate dehydrogenase; GABA, γ-aminobutyric acid; GDNF, glial cell line-derived neurotrophic factor; GPX4, glutathione peroxidase 4; GSH, glutathione; HT, hemorrhagic transformation; IL, interleukin; IV, intravenous; LNPs, lipid nanoparticles; M1/M2, conventional labels for classically/alternatively activated myeloid states; miRNAs, microRNAs; MMP, matrix metalloproteinase; MMP-9, matrix metalloproteinase-9; mtDNA, mitochondrial DNA; NADPH, reduced nicotinamide adenine dinucleotide phosphate; NETs, neutrophil extracellular traps; NF-κB, nuclear factor kappa B; NK, natural killer; NLRP3, NOD-like receptor family pyrin domain-containing 3; NMDAR, N-methyl-D-aspartate receptor; NRF2, nuclear factor erythroid 2-related factor 2; O_2_•−, superoxide anion; OPC, oligodendrocyte precursor cell; OXPHOS, oxidative phosphorylation; PINK1, PTEN-induced kinase 1; PPAR, peroxisome proliferator-activated receptor; PPP, pentose phosphate pathway; ROS, reactive oxygen species; SDH, succinate dehydrogenase; STAIR, Stroke Therapy Academic Industry Roundtable; TCA, tricarboxylic acid cycle; TGF-β, transforming growth factor beta; TIMP-1, tissue inhibitor of metalloproteinases-1; TNF-α, tumor necrosis factor alpha; TREM2, triggering receptor expressed on myeloid cells 2; Treg, regulatory T cell; VWF, von Willebrand factor; ZO-1, zonula occludens-1; ΔΨm, mitochondrial membrane potential.

During the chronic phase, immune responses further transition toward homeostatic maintenance and functional recovery. Lymphocyte populations, including T cells, Tregs, B cells, and natural killer (NK) cells, undergo stage-specific changes and influence long-term outcomes by regulating inflammatory resolution, white-matter repair, and neurovascular remodeling (165). Endothelial cells can also regulate lymphocyte transvascular migration through reactive vascular structures, indicating that immune time windows are not determined solely by immune cells themselves but are also shaped by dynamic changes in the vascular microenvironment (165). Concomitantly, lactate, lactylation, and mitochondrial damage-associated signals can persistently modulate immune-cell function. As a result, chronic inflammation may either support reparative remodeling or evolve into sustained low-grade inflammation, depending on the prevailing metabolic and microenvironmental context (14, 166) (**Figure 3**).

## 5 Metabolic reprogramming across substrates, cells, and organs

### 5.1 Glucose, glycolysis, lactate, and the pentose phosphate pathway

Post-ischemic glucose and lactate metabolism must be interpreted at the level of specific cell types and compartments. Neurons have high oxidative demands but limited energy reserves, whereas astrocytes can increase glycolysis, store glycogen, take up glutamate, and export lactate through monocarboxylate transporters (MCTs). Activated microglia and infiltrating myeloid cells also undergo state-dependent changes in glycolysis, the pentose phosphate pathway, mitochondrial respiration, and lipid utilization. Oligodendrocytes provide metabolic support to axons through monocarboxylate transport and are particularly sensitive to energetic failure (167). An increase in bulk-tissue lactate therefore cannot by itself distinguish maladaptive acidosis from compensatory substrate shuttling.

After IS, insufficient oxygen and glucose supply restricts mitochondrial oxidative phosphorylation, forcing neurons and glial cells to enhance glycolysis to sustain basal ATP production. Increased lactate generation is a hallmark of this metabolic reprogramming. Lactate accumulation reflects local bioenergetic stress and can reshape the microenvironment of the ischemic penumbra (168). Excessive lactate accumulation may induce local acidification and impose additional metabolic burden, whereas moderate lactate availability can serve as both an energy substrate and a signaling molecule involved in ischemic adaptation. Thus, the biological effects of lactate are highly dependent on its concentration, temporal window, and cellular context (169).

Lactate functions as a metabolic substrate, signaling molecule, and epigenetic precursor after stroke. Its substrate role depends on cellular source, transporter directionality, tissue oxygenation, and preserved oxidative capacity, as discussed in *Section 6.1*. In microglia, lactate can modulate hypoxia-inducible factor 1 alpha (HIF-1α)-related inflammatory signaling and drive histone or non-histone lactylation. Histone H3 lysine 18 lactylation (H3K18la)-associated Plxnb2 signaling has been linked to attenuated neuroinflammation, whereas increased histone H3 lysine 9 lactylation (H3K9la) has been associated with proinflammatory gene expression (169–173). These divergent findings argue against treating bulk-tissue lactate as uniformly protective or harmful.

Enhanced glycolysis also affects immune-cell function. Single-cell studies have shown that microglia after ischemia exhibit increased glycolytic activity, suppression of the tricarboxylic acid (TCA) cycle, and altered lipid metabolism. On the one hand, this metabolic state can promote the transcription of inflammation-associated genes and the release of proinflammatory cytokines, including tumor necrosis factor-α (TNF-α) and interleukin-1β (IL-1β), thereby strengthening microglial inflammatory responses. On the other hand, disrupted oxidative and lipid metabolism alters the ability of microglia to recognize and clear cellular debris, necrotic tissue, and synaptic components, ultimately influencing phagocytic function and post-injury tissue remodeling (87). Collectively, enhanced glycolysis and altered lactate metabolism constitute a key metabolic node that connects energy compensation, inflammatory regulation, and epigenetic remodeling after IS.

### 5.2 Mitochondrial substrates, redox balance, and ketone bodies

Mitochondrial dysfunction is a central component of metabolic reprogramming after IS. Ischemia restricts oxygen and glucose availability, impairs the mitochondrial respiratory chain, reduces ATP production, and is accompanied by loss of mitochondrial membrane potential, disruption of Ca^2+^ homeostasis, and excessive generation of reactive oxygen species (ROS). These alterations not only compromise neuronal energy maintenance but also activate inflammatory responses through mitochondria-derived damage signals, including mitochondrial DNA (mtDNA) and ROS.

Mitophagy is a key mechanism of mitochondrial quality control. Stroke-focused experimental work indicates that PINK1/Parkin/p62-dependent mitophagy is engaged after cerebral ischemia–reperfusion (174). Its net effect is not necessarily uniform: the balance between removal of damaged organelles and preservation or replacement of the functional mitochondrial pool depends on injury severity, cell type, and phase; excessive or sustained organelle removal may deplete functional mitochondria rather than restore bioenergetic reserve (174, 175).

Alternative mitochondrial substrates may become relevant when glucose oxidation is impaired. Ketone bodies, particularly β-hydroxybutyrate, can serve as oxidative substrates and signaling metabolites. In rodent ischemic-stroke models, ketone administration improved mitochondrial function and reduced oxidative stress through SIRT3-related signaling (176), and β-hydroxybutyrate reduced injury through ERK/CREB/eNOS-associated mitochondrial and antioxidant effects (177). These findings remain preclinical and do not establish an effective dose, route, or treatment window in patients. Ketone metabolism therefore illustrates substrate flexibility rather than a clinically validated therapy.

### 5.3 Lipid mobilization, lipid peroxidation, and ferroptotic stress

After IS, lipid homeostasis in the brain is disrupted early by myelin injury, membrane rupture, and local disturbances in lipoprotein metabolism. Myelin sheaths and cellular membranes are enriched in cholesterol, phospholipids, sphingolipids, and fatty acids, which are essential for axonal conduction, synaptic architecture, and membrane integrity. Ischemia–hypoxia and reperfusion injury promote membrane lipid peroxidation, myelin breakdown, and the release of lipid-rich debris, thereby creating a high-lipid-burden microenvironment. Phospholipid remodeling can also influence mitochondrial membrane dynamics and bioenergetic behavior, linking lipid disequilibrium to organelle-level metabolic vulnerability (178). These lipid species are not merely by-products of tissue damage; they also act as bioactive signals that shape the metabolic state and inflammatory behavior of phagocytes.

During lipid handling after stroke, microglia and MDMs are the principal cellular populations responsible for lipid uptake, degradation, and reutilization. TREM2 signaling promotes microglial recognition of lipid-associated damage signals, enhances phagocytosis, and regulates lipid metabolism and cell survival, making it an important molecular node in the post-stroke response to lipid burden (89). Single-cell studies further indicate that post-stroke phagocytes upregulate genes related to lipid processing, cholesterol metabolism, and lysosomal function, reflecting metabolic adaptation to myelin- and membrane-derived debris. In this context, activating transcription factor 3 (ATF3) can regulate cholesterol 25-hydroxylase (CH25H) expression, thereby linking cholesterol hydroxylation metabolism to inflammatory mediator production and suggesting that altered cholesterol metabolism can directly influence immune activity (87). In addition, peroxisome proliferator-activated receptor (PPAR)-related signaling participates in fatty acid oxidation, cholesterol efflux, and inflammatory suppression. The functional state of this pathway may determine whether phagocytes remain primarily debris-clearing cells or transition toward a reparative metabolic phenotype (11, 179).

Lipid droplets are neutral-lipid storage organelles and metabolic buffers that can transiently sequester excess fatty acids and cholesteryl esters (180). When phagocytes continuously engulf myelin-rich debris but cannot complete lipid processing, lipid-laden or foam-like cells can accumulate. In male and female mice, abundant lipid-rich foam cells persisted within chronic infarcts 30 days after transient MCAO (181). Impaired lipid handling has been proposed to sustain inflammatory signaling, while microglial AXL deletion increased delayed lipid-droplet accumulation, myelin-debris retention, and white-matter injury (182, 183). These observations link defective phagocyte lipid processing to chronic inflammation and impaired myelin repair in preclinical models, but the mechanism has not yet been established directly in patients.

Beyond microglia and macrophages, astrocytic lipid metabolism also contributes to local repair after stroke. One experimental study using the MMP-9 inhibitor SB-3CT suggests that modulation of MMP-9 activity can influence astrocytic cholesterol, sphingolipid, and glycerophospholipid metabolism, reduce the accumulation of neurotoxic lipids, and support neuronal survival and synaptic integrity (184). Nevertheless, this evidence primarily highlights the indirect impact of MMP-9-targeted intervention on lipid metabolism rather than establishing astrocytic lipid remodeling as an isolated therapeutic mechanism.

Clinical lipid associations do not map linearly onto experimental lipotoxicity. Oxidized phospholipids, free fatty acid accumulation, and enzymatic lipid peroxidation can amplify inflammation and ferroptotic injury within ischemic tissue. At the clinical level, observational studies have reported better short-term outcome among patients with higher cholesterol or triglyceride concentrations, a phenomenon described as a lipid paradox (185). Conversely, lower cholesterol has been associated with greater risk of hemorrhagic transformation in some thrombolysis cohorts (186, 187). These associations may reflect nutritional status, frailty, stroke subtype, prior statin exposure, recanalization treatment, and reverse causation rather than a protective effect of dyslipidemia.

The lipid paradox should not be interpreted as an argument against lipid-lowering therapy for secondary prevention. In the Stroke Prevention by Aggressive Reduction in Cholesterol Levels trial, high-dose atorvastatin reduced recurrent stroke and cardiovascular events in selected patients with recent stroke or transient ischemic attack, although hemorrhagic-stroke risk required consideration (188). Acute circulating lipid associations, tissue lipid-peroxidation mechanisms, and long-term vascular risk modification therefore represent distinct biological and clinical questions.

### 5.4 Amino-acid metabolism, neurotransmitter cycling, and one-carbon metabolism

After IS, disruption of amino-acid metabolism is first manifested by impaired glutamate homeostasis. Energy depletion compromises glutamate uptake and transport in astrocytes and neurons, leading to glutamate accumulation in the synaptic cleft and extracellular space. Excessive glutamate persistently activates NMDARs, triggering Ca^2+^ influx, increased mitochondrial burden, and excitotoxic injury, thereby aggravating neuronal damage (16). Concomitantly, non-neuronal cells also participate in the regulation of glutamate homeostasis. Microglia, astrocytes, and vascular-associated cells can influence local excitotoxic responses through glutamate release, uptake, and receptor-mediated signaling (189). These alterations underscore the fundamental role of amino-acid metabolism in maintaining neurotransmitter homeostasis after IS.

Alterations in inhibitory neurotransmission further exacerbate the excitatory–inhibitory imbalance. After ischemia, impaired GABA-related signaling weakens inhibitory regulation within neural networks, rendering neurons more vulnerable to excessive excitatory stimulation. Studies targeting the downregulation of GABA_B receptors suggest that preservation of inhibitory neurotransmission may reduce neuronal vulnerability under ischemic conditions (190). Thus, glutamate-driven hyperexcitation and insufficient GABAergic inhibition jointly constitute major features of post-stroke neurotransmitter imbalance.

Beyond classical excitatory and inhibitory neurotransmitter systems, arginine and tryptophan metabolism also contribute to post-stroke metabolic regulation. Arginine can be converted by nitric oxide synthase (NOS) into nitric oxide (NO), which participates in vasodilation and redox regulation. However, under conditions of enhanced oxidative stress, NO-related pathways may interact with ROS, thereby aggravating nitrative stress. Clinical evidence indicates that elevated asymmetric dimethylarginine (ADMA) is associated with suppressed NO signaling and impaired leptomeningeal collateral circulation, suggesting that the arginine–NO axis may influence perfusion adaptation after ischemia (191). The tryptophan–kynurenine pathway generates metabolites with divergent biological effects. Some metabolites participate in neuroprotection and immune regulation, whereas quinolinic acid and related metabolites may promote oxidative stress and neurotoxicity (192). In addition, gut microbiota-derived indole tryptophan metabolites, including indole-3-propionic acid, have antioxidant, immunomodulatory, and neuroprotective potential, suggesting that tryptophan metabolism may regulate post-stroke inflammation and oxidative stress through the gut–brain axis (193). Overall, amino-acid metabolic remodeling provides an important basis for post-ischemic neurotransmitter homeostasis and metabolic adaptation by modulating excitatory–inhibitory balance, arginine–NO signaling, and the tryptophan metabolite profile.

### 5.5 Regulated cell death as an inflammatory–metabolic interface

Regulated cell-death programs provide a mechanistic interface between energy failure, mitochondrial dysfunction, redox imbalance, membrane damage, and inflammation after IS. Apoptosis and unregulated necrosis remain relevant, but ferroptosis, necroptosis, and pyroptosis explain distinct components of lipid-peroxidative, kinase-dependent, and inflammasome-driven injury. These pathways can interact rather than operate in isolation. Cuproptosis is a more recently defined copper-dependent mitochondrial death program, but its causal contribution to cerebral ischemia is less established and is treated here as emerging evidence.

#### 5.5.1 Ferroptosis

Ferroptosis is an iron-dependent form of regulated cell death driven by unchecked phospholipid peroxidation. It was defined as a distinct regulated-death program in 2012 (194). Loss of glutathione-dependent glutathione peroxidase 4 (GPX4) activity permits toxic lipid hydroperoxide accumulation (195), while acyl-CoA synthetase long-chain family member 4 (ACSL4) promotes incorporation of ferroptosis-susceptible polyunsaturated fatty acids into membrane phospholipids (196). Cerebral ischemia creates a permissive environment through glutathione depletion, mitochondrial and reduced nicotinamide adenine dinucleotide phosphate (NADPH) stress, iron dysregulation, and enzymatic lipid oxidation. In experimental stroke, tau-dependent iron export was linked to protection from ferroptotic injury (197). The core iron–phospholipid–GPX4 axis distinguishes ferroptosis from nonspecific oxidative neuronal death. Ferroptosis inhibitors, lipid-radical-trapping antioxidants, and iron-handling strategies have shown preclinical effects, but clinical efficacy and compatibility with reperfusion therapy remain unestablished.

#### 5.5.2 Necroptosis

Necroptosis is a lytic, kinase-regulated death program centered on receptor-interacting serine/threonine-protein kinase 1 (RIPK1), receptor-interacting serine/threonine-protein kinase 3 (RIPK3), and mixed lineage kinase domain-like pseudokinase (MLKL). The discovery that necrostatin-1 could inhibit a regulated necrotic pathway and reduce ischemic brain injury provided early evidence that necrosis can be pharmacologically modulated (198). Downstream, RIPK3-dependent activation and oligomerization of MLKL disrupt plasma-membrane integrity and release damage-associated signals (199). Experimental cerebral ischemia studies have linked RIPK1/RIPK3/MLKL signaling to tissue injury and inflammatory amplification (200), and more recent MLKL-directed approaches remain preclinical (201). Necroptosis may be favored when caspase-8-dependent apoptosis is constrained, emphasizing the importance of pathway context. Although RIPK1, RIPK3, and MLKL are mechanistically attractive targets, intervention may alter host defense and inflammatory signaling; temporal and cell-specific validation is required before clinical translation.

#### 5.5.3 Pyroptosis

Pyroptosis is an inflammatory lytic death program executed by gasdermin pores. Canonical inflammasome activation promotes caspase-1-dependent cleavage of GSDMD, pore formation, cell swelling, and release of IL-1β and IL-18; landmark studies identified GSDMD as a central executioner of pyroptosis (202, 203). In IS, NLRP3, AIM2, and cGAS–STING-related signals may converge on inflammasome activation in microglia, infiltrating myeloid cells, endothelial cells, and other NVU populations. In experimental stroke, the caspase-1 inhibitor VX-765 reduced inflammatory injury (204), and direct suppression of GSDMD-mediated pyroptosis has shown preclinical benefit (205). Inflammasome activation may produce cytokines without immediate cell lysis; therefore, NLRP3 activation and pyroptotic death are related but not synonymous.

#### 5.5.4 Cuproptosis

Cuproptosis is a copper-dependent death program linked to mitochondrial protein lipoylation. The defining study showed that excess intracellular copper binds lipoylated components of the tricarboxylic acid cycle machinery, promotes aggregation of lipoylated proteins and loss of iron–sulfur-cluster proteins, and induces proteotoxic stress; ferredoxin 1 (FDX1) and the protein-lipoylation machinery are central determinants (206). This mechanism is conceptually relevant to ischemic tissue because mitochondrial metabolism, redox balance, and metal homeostasis are profoundly disturbed. Evidence for cuproptosis in cerebral ischemia is currently less direct than evidence for ferroptosis, necroptosis, or pyroptosis. Recent ischemic-stroke studies have largely used transcriptomic datasets, machine-learning-defined gene clusters, or biomarker prediction (207, 208), and reviews have proposed interactions between copper homeostasis, ferroptosis, and ischemic injury (209). Cuproptosis should therefore be regarded as an emerging hypothesis until copper-dependent, FDX1/lipoylation-dependent death is demonstrated in defined ischemic brain cells.

#### 5.5.5 Cell-death crosstalk and PANoptosis

The regulated cell-death pathways described above should not be interpreted as mutually exclusive terminal labels. In a reperfused mouse stroke model, transcriptomic and protein analyses placed ferroptotic and necroptotic signals early after reperfusion, followed by apoptotic features; ferroptosis and necroptosis inhibitors also showed partial cross-suppression, and iron loading promoted both pathways (210). This supports a shared redox–iron–membrane-injury network in which blocking one execution program may redirect or attenuate another. It also helps explain why single-pathway inhibitors can show model- and time-dependent effects. PANoptosis describes coordinated engagement of pyroptotic, apoptotic, and necroptotic machinery within an integrated inflammatory death response. Experimental work in rats and primary neurons reported that vagus-nerve stimulation reduced molecular features assigned to neuronal PANoptosis through Sirt1-dependent signaling and improved recovery (211). However, simultaneous detection of several pathway markers does not by itself prove formation of a single PANoptosome or identify the dying cell population. In stroke, PANoptosis should therefore be presented as a testable integrative framework, with causal validation requiring cell-resolved perturbation of NVU components, temporal ordering, and demonstration that combined pathway control outperforms selective inhibition.

### 5.6 Cell-specific metabolic states and aging-related vulnerability within the NVU

Metabolic reprogramming is both a consequence and a regulator of inflammatory cell state. Glycolytic activation can rapidly support cytokine production and phagocytic activity, whereas oxidative phosphorylation, fatty-acid oxidation, and mitochondrial quality control may support selected reparative programs. However, equating glycolysis with a harmful M1 state and oxidative metabolism with a beneficial M2 state is inadequate. Microglia, macrophages, astrocytes, endothelial cells, and OPCs display mixed and time-dependent transcriptional and metabolic states. Measured pathways and functions, such as glycolytic flux, mitochondrial respiration, lipid-droplet accumulation, ROS generation, or efferocytosis, are more informative than a binary metabolic phenotype.

Cellular metabolic roles within the NVU are complementary but not interchangeable. Neurons depend strongly on oxidative metabolism and have limited intrinsic reserves; astrocytes provide glycolytic, glycogen-derived, antioxidant, and neurotransmitter-clearing support; oligodendrocytes supply axons with monocarboxylate substrates; and microglial, endothelial, and pericyte metabolism changes with inflammatory state, barrier demand, and perfusion. Neuron–astrocyte lactate transport, its bidirectional effects, and its therapeutic implications are considered at the interaction-network level in *Section 6.1* rather than repeated here (212, 213).

Aging may diminish the functional reserve of the multicellular energy network before stroke and reduce its capacity to adapt to ischemic stress. Disturbances in mitochondrial biogenesis, fusion–fission balance, mitophagy, and intercellular mitochondrial communication may create a cell-specific energy crisis across the NVU. Within this framework, senescent astrocytes may impair astrocyte–neuron lactate transfer, oligodendrocyte ATP deficiency may compromise axonal and myelin support, and dynamin-related protein 1-associated mitochondrial fission may promote maladaptive microglial immunometabolism. Intercellular mitochondrial transfer may partially counteract these deficits (214). These mechanisms provide an aging-related vulnerability framework, but their contribution to stroke progression requires direct validation in aged stroke models and appropriately stratified human cohorts.

### 5.7 Systemic metabolic responses and comorbidities

Stroke also induces systemic metabolic remodeling. Catecholamine and glucocorticoid release promote hepatic glucose production, adipose lipolysis, and insulin resistance, while reduced intake, immobility, infection, and pre-existing diabetes further alter substrate availability. Stress hyperglycemia, especially when expressed relative to chronic glycemic status, has been associated with greater neurological deficit and worse outcome in patients with acute IS (215). Peripheral lipid, amino-acid, and ketone metabolism may likewise influence immune-cell function and brain substrate supply, but causal human data remain limited.

Systemic metabolic status influences post-stroke remodeling by modifying vascular reserve, immune responses, and cellular metabolism. Diabetes and stress hyperglycemia can aggravate oxidative stress, BBB disruption, and microcirculatory dysfunction, but chronic diabetes and an acute stress response are distinct biological and clinical contexts (216, 217). Dyslipidemia, hypertension, and aging also modify endothelial function and immune-metabolic reserve; their effects on repair vary across models, reperfusion histories, and patient populations (216–219).

Systemic metabolic factors can reshape neurovascular–immune function through interacting transcriptional, epigenetic, and noncoding-RNA programs. Diabetes and hyperglycemia are associated with endothelial dysfunction, oxidative stress, and inflammatory activation, but effect size and direction vary with sex, chronic glycemic exposure, and reperfusion context (217). Chronic phagocyte lipid-handling failure, including the AXL-associated lipid-droplet and foam-cell findings, is detailed in *Section 5.3*. At the systemic level, these local mechanisms illustrate how pre-existing metabolic state and peripheral substrate availability may condition the inflammatory and reparative capacity of the post-stroke network.

### 5.8 Metabolites as immune signals and redox regulators

Tricarboxylic acid cycle intermediates can function as signals rather than merely as fuel. In activated macrophages, succinate stabilizes HIF-1α and promotes IL-1β production (220). In ischemia, succinate also accumulates during oxygen deprivation and can be rapidly oxidized by succinate dehydrogenase (SDH) at reperfusion, driving mitochondrial reactive oxygen species. Human and mouse brain tissue exposed to ischemia *ex vivo* showed time-dependent succinate accumulation, and peri-reperfusion malonate reduced injury in a mouse mechanical-thrombectomy model (221). This provides a mechanistic bridge between metabolic substrate accumulation and reperfusion injury, while remaining preclinical as a treatment strategy.

SDH may also connect metabolism to specific inflammatory and death programs. Experimental studies reported that TIGAR limited neuronal ferroptosis by suppressing succinate dehydrogenase activity (222) and that succinate dehydrogenase subunit A-dependent succinate oxidation promoted microglial extracellular-trap formation after ischemia–reperfusion (223). These findings suggest that the same enzymatic node can influence neuronal lipid peroxidation and myeloid effector function. Whether a clinically usable intervention can inhibit injurious reverse electron transport without compromising mitochondrial recovery requires pharmacokinetic, cell-specific, and reperfusion-compatible validation.

**Figure 4:**
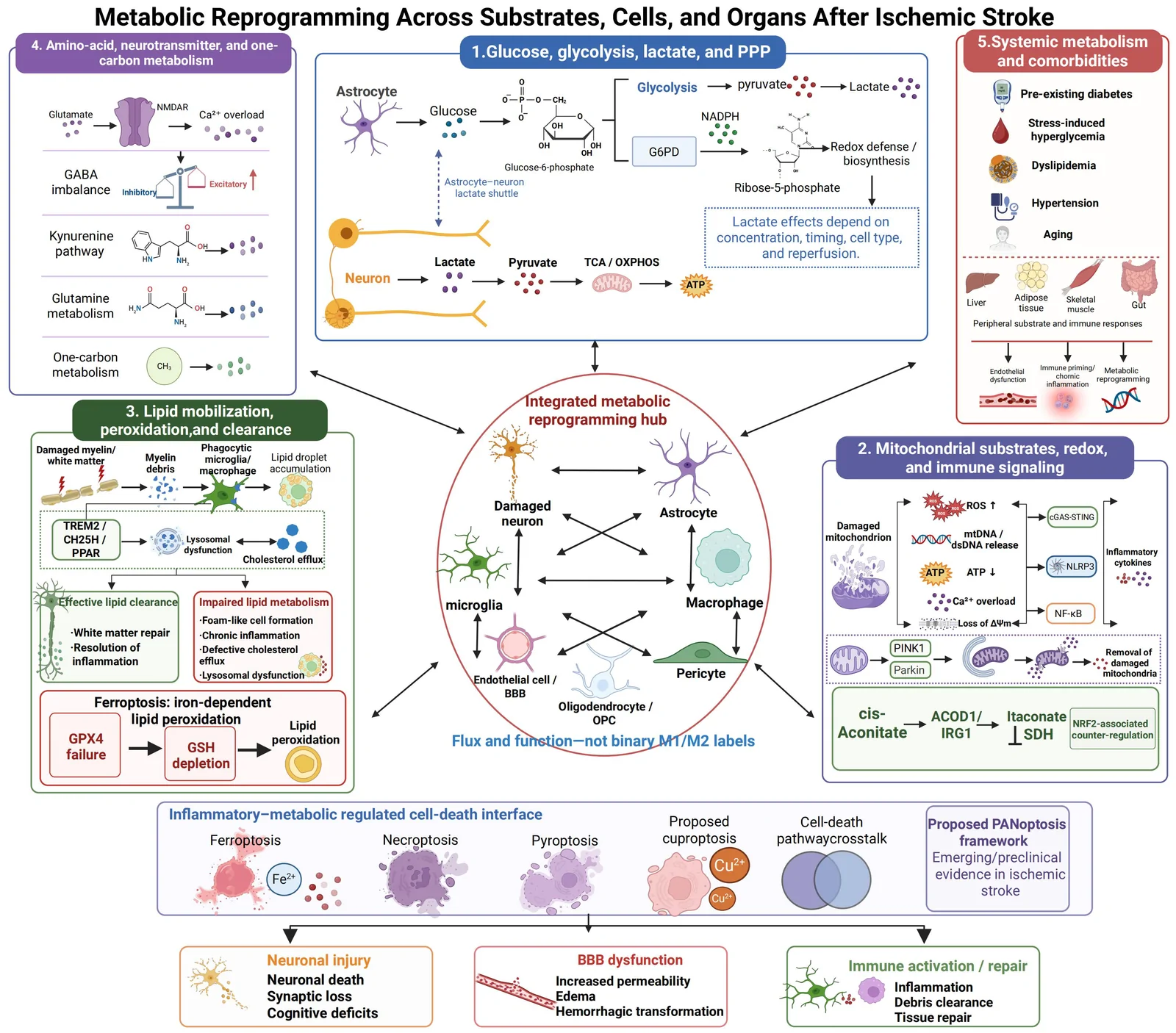
Metabolic reprogramming across substrates, cells, and organs after ischemic stroke. The central integrated metabolic-reprogramming hub places damaged neurons, astrocytes, microglia, macrophages, endothelial cells/the BBB, pericytes, and oligodendrocytes/OPCs within an interconnected network, emphasizing measured metabolic flux and function rather than binary M1/M2 labels. Module 1 shows astrocytic glucose utilization, formation of glucose-6-phosphate, glycolysis to pyruvate and lactate, and the G6PD-dependent pentose-phosphate pathway producing NADPH and ribose-5-phosphate for redox defense and biosynthesis. The astrocyte–neuron lactate shuttle connects this module to neuronal conversion of lactate to pyruvate and ATP generation through the TCA cycle and OXPHOS; the effect of lactate is explicitly conditioned by concentration, timing, cell type, and reperfusion. Module 2 links a damaged mitochondrion to increased ROS, mtDNA/dsDNA release, decreased ATP, Ca^2+^ overload, and loss of ΔΨm, which are connected to cGAS–STING, NLRP3, NF-κB, and inflammatory-cytokine signaling. PINK1/Parkin-dependent mitophagy is shown as a route for removing damaged mitochondria, whereas the cis-aconitate–ACOD1/IRG1–itaconate branch depicts SDH inhibition and NRF2-associated counter-regulation. Module 3 follows damaged myelin/white matter through myelin-debris uptake by phagocytic microglia/macrophages and lipid-droplet accumulation and groups TREM2-, CH25H-, PPAR-, lysosome-, and cholesterol-efflux-related lipid handling. Effective lipid clearance is associated with white-matter repair and resolution of inflammation, whereas impaired lipid metabolism is associated with foam-like cell formation, chronic inflammation, defective cholesterol efflux, and lysosomal dysfunction. The ferroptosis inset groups GPX4 failure, GSH depletion, and iron-dependent lipid peroxidation. Module 4 depicts glutamate–NMDAR–Ca^2+^-overload signaling, excitatory/inhibitory imbalance involving GABA, and the kynurenine, glutamine, and one-carbon metabolic pathways. Module 5 separates pre-existing diabetes from stress-induced hyperglycemia and includes dyslipidemia, hypertension, and aging; liver, adipose tissue, skeletal muscle, and gut responses are connected to peripheral substrate and immune responses, endothelial dysfunction, immune priming/chronic inflammation, and metabolic reprogramming. The lower band presents ferroptosis, necroptosis, pyroptosis, proposed cuproptosis, cell-death-pathway crosstalk, and a proposed PANoptosis framework as an inflammatory–metabolic regulated-cell-death interface leading to neuronal injury, BBB dysfunction, and immune activation or repair. Single-headed arrows indicate the direction of reactions or processes as drawn; double-headed arrows indicate reciprocal cellular or module-level coupling; upward and downward arrows indicate increases and decreases, respectively; and blunt-ended lines indicate inhibition. Dotted outlines group mechanistic submodules or contextual qualifiers, and “proposed” denotes emerging/preclinical status in ischemic stroke. ACOD1/IRG1, aconitate decarboxylase 1/immune-responsive gene 1; ATP, adenosine triphosphate; BBB, blood–brain barrier; BDNF, brain-derived neurotrophic factor; Ca^2+^, calcium ion; cfDNA, cell-free DNA; cGAS–STING, cyclic GMP–AMP synthase–stimulator of interferon genes; CH_3_, methyl group; CH25H, cholesterol 25-hydroxylase; Cu^2+^, copper(II) ion; DAMPs, damage-associated molecular patterns; dsDNA, double-stranded DNA; ECM, extracellular matrix; EVs, extracellular vesicles; Fe^2+^, ferrous ion; G6PD, glucose-6-phosphate dehydrogenase; GABA, γ-aminobutyric acid; GDNF, glial cell line-derived neurotrophic factor; GPX4, glutathione peroxidase 4; GSH, glutathione; HT, hemorrhagic transformation; IL, interleukin; IV, intravenous; LNPs, lipid nanoparticles; M1/M2, conventional labels for classically/alternatively activated myeloid states; miRNAs, microRNAs; MMP, matrix metalloproteinase; MMP-9, matrix metalloproteinase-9; mtDNA, mitochondrial DNA; NADPH, reduced nicotinamide adenine dinucleotide phosphate; NETs, neutrophil extracellular traps; NF-κB, nuclear factor kappa B; NK, natural killer; NLRP3, NOD-like receptor family pyrin domain-containing 3; NMDAR, N-methyl-D-aspartate receptor; NRF2, nuclear factor erythroid 2-related factor 2; O_2_•−, superoxide anion; OPC, oligodendrocyte precursor cell; OXPHOS, oxidative phosphorylation; PINK1, PTEN-induced kinase 1; PPAR, peroxisome proliferator-activated receptor; PPP, pentose phosphate pathway; ROS, reactive oxygen species; SDH, succinate dehydrogenase; STAIR, Stroke Therapy Academic Industry Roundtable; TCA, tricarboxylic acid cycle; TGF-β, transforming growth factor beta; TIMP-1, tissue inhibitor of metalloproteinases-1; TNF-α, tumor necrosis factor alpha; TREM2, triggering receptor expressed on myeloid cells 2; Treg, regulatory T cell; VWF, von Willebrand factor; ZO-1, zonula occludens-1; ΔΨm, mitochondrial membrane potential.

Itaconate provides a counter-regulatory example. Foundational studies showed that macrophage-derived itaconate restrains succinate dehydrogenase activity and inflammatory cytokine production (224) and that electrophilic itaconate signaling can activate NRF2 through Kelch-like ECH-associated protein 1 alkylation (225). These mechanisms are biologically relevant to post-stroke myeloid remodeling, but direct human stroke evidence is not yet sufficient to treat itaconate derivatives as established therapy. Aging introduces an additional metabolic constraint: NAD+ depletion was associated with hyperinflammatory monocytes and gut-barrier dysfunction after stroke in aged mice, whereas nicotinamide riboside pretreatment normalized several immune and outcome measures (98). Because treatment was preventive in that study, efficacy after clinical stroke onset remains unresolved (**Figure 4**).

## 6 Neurovascular–immune–metabolic interaction networks

### 6.1 Neuron–astrocyte metabolic coupling

Neurons have continuously high energy demands but limited intrinsic reserves, whereas astrocytes are positioned between microvessels and synapses, possess strong glycolytic capacity, and can store glycogen. Under physiological conditions, astrocytic glutamate uptake increases Na+/K+-ATPase activity, glucose utilization, glycolysis, and lactate production, providing an experimental basis for the astrocyte–neuron lactate-shuttle hypothesis (226). After IS, astrocytes can temporarily support energy metabolism through glycogenolysis, glucose transporter 1-mediated glucose uptake, and glycolysis. Lactate exported through MCT1 and MCT4 can be taken up through neuronal MCT2 and oxidized when residual mitochondrial capacity or reperfusion is sufficient (227, 228). This transport is governed by substrate and proton gradients, transporter expression, tissue pH, and oxidative capacity; it is not a fixed unidirectional process.

The protective effects of this coupling extend beyond lactate delivery. Astrocytes require glycolytically generated ATP to sustain glutamate uptake, Na^+^/K^+^-ATPase activity, potassium buffering, and the glutamate–glutamine cycle, thereby limiting excitotoxic injury. A proportion of astrocytic glucose also enters the pentose phosphate pathway, generating NADPH and supporting glutathione-dependent antioxidant systems that help protect neurons against the reactive oxygen species burden generated during ischemia and reperfusion (213). Increased astrocytic glycolysis during early ischemia may therefore represent a compensatory response. Without considering timing, cellular state, and tissue oxygenation, indiscriminate inhibition of astrocytic glycolysis could simultaneously weaken glutamate clearance, ionic homeostasis, and neuronal energy support. Conversely, when ischemia persists and astrocytic mitochondria and membrane-transport systems become dysfunctional, this compensatory network progressively fails, allowing glutamate, lactate, ROS, and inflammatory mediators to accumulate concurrently and accelerate neuronal energy failure.

The effects of lactate in stroke are strongly time-dependent. In mouse transient middle cerebral artery occlusion models, lactate administered at the onset of reperfusion reduced tissue injury and improved neurological outcomes, suggesting that lactate can serve as an alternative neuronal substrate when oxidative metabolism has partially recovered (229). Some of its neuroprotective activity may also involve hydroxycarboxylic acid receptor 1 (HCAR1/GPR81) signaling rather than metabolic oxidation alone (230). Nevertheless, these findings are derived primarily from rodent experiments, and the effects of lactate treatment can vary according to the anesthetic regimen, treatment timing, circulating lactate concentration, and reperfusion status (231). Exogenous lactate should therefore not be presented as a clinically validated treatment for IS.

By contrast, during ongoing ischemia before reperfusion, sustained glycolytic lactate production may exceed the uptake and oxidative capacity of surrounding cells. A mouse stroke study showed that lactate accumulating during the ischemic phase, primarily from astrocytes, promoted widespread protein lactylation and aggravated brain injury, whereas inhibition of lactate dehydrogenase A or glycolysis attenuated the corresponding damage (232). Through histone and non-histone protein lactylation, lactate can also translate metabolic status into changes in transcription and protein function, thereby affecting neuronal death, microglial inflammatory responses, endothelial-barrier integrity, and tissue repair (166, 233). An increase in lactate should therefore not be interpreted uniformly as either protective metabolic support or harmful acidosis. Its net effect depends on cellular source, uptake capacity, subcellular targets, tissue pH, oxygen availability, and whether the tissue remains ischemic or has entered reperfusion.

Neuron–astrocyte metabolic coupling also reshapes the local immune microenvironment. Efficient lactate transport and oxidation can preserve neuronal viability and reduce upstream DAMP release, whereas extracellular lactate and H+ accumulation can interact with ROS, ATP, high mobility group box 1 (HMGB1), and cytokines to reinforce metabolic–inflammatory stress. Reactive astrocyte populations may combine altered glycolysis, mitochondrial suppression, antioxidant activity, and inflammatory-mediator release in different proportions (212). Accordingly, a more informative experimental target or readout is the efficiency and direction of coupling rather than absolute lactate concentration. Future studies should integrate cell-specific flux, MCT1/MCT4/MCT2 expression, lactate-to-pyruvate ratios, tissue pH, mitochondrial oxidative capacity, and reperfusion across the hyperacute, acute, subacute, and chronic phases. Age, diabetes, and collateral status may further modify this network.

### 6.2 Metabolic–microvascular feedback and no-reflow

Under ischemic brain injury, the vascular coupling network formed by astrocytes, endothelial cells, and pericytes is essential for maintaining cerebral microcirculation and BBB function. Astrocytic endfeet closely ensheath cerebral microvessels and directly support endothelial cells in preserving tight junction integrity, selective barrier permeability, and blood-flow dynamics. Endothelial cells not only constitute the core structural basis of the BBB but also coordinate the balanced transport of blood flow, nutrients, and metabolic waste by regulating ion channels, transporters, and intercellular junctional proteins, including tight junction and adherens junction components (63, 234). Pericytes are embedded within the vascular basement membrane and fine-tune capillary tone through contractile and relaxant responses, thereby contributing to local blood-flow regulation and maintaining microvascular homeostasis through bidirectional signaling with endothelial cells (5, 235).

Ischemia-induced reactive changes in astrocytes not only increase the secretion of proinflammatory mediators but also promote abnormal accumulation of local signaling metabolites. For example, excessive lactate accumulation in the ischemia–reperfusion microenvironment enhances histone H3K18la in BMECs, thereby activating the activating transcription factor 4/DNA damage-inducible transcript 4 (ATF4/DDIT4) signaling pathway, suppressing mitochondrial function, and promoting RIPK3-dependent endothelial cell death. This self-reinforcing metabolic–epigenetic feedback loop directly compromises endothelial barrier integrity (236, 237). In addition, the early burst of ROS after ischemia induces profound oxidative stress, leading to degradation of endothelial tight junction proteins and increased barrier permeability (238).

Concomitantly, HMGB1 and extracellular ATP released from injured cells can act as damage-associated signals at the neurovascular interface. Experimental cerebral ischemia links HMGB1 release to BBB disruption (239), whereas extracellular ATP induced P2Y-dependent Ca^2+^ signaling and contraction in cultured brain capillary pericytes. The latter finding establishes *in vitro* mechanistic plausibility but is not direct evidence that ATP drives no-reflow *in vivo*. *In vivo* studies more directly link persistent pericyte constriction and dysfunction after ischemia–reperfusion to impaired capillary reflow (72, 240). MiRNAs and EVs further mediate bidirectional signaling among glia, endothelium, and pericytes and may participate in barrier repair (241).

### 6.3 Vascular–immune and thromboinflammatory coupling

After ischemia, brain endothelial cells become active inflammatory signaling hubs rather than passive barrier elements. Endothelial transcriptomic and translatomic profiling 24 h after experimental stroke identified programs related to leukocyte attraction, adhesion, BBB leakage, and vascular remodeling (242). Human post-mortem tissue likewise showed marked ICAM-1 upregulation on infarct-region microvessels, temporally associated with granulocyte accumulation. In mouse ischemia–reperfusion, rapid ROCK/MLC-dependent endothelial cytoskeletal reorganization disrupted junctional organization and facilitated later entry of MMP-9-producing leukocytes (243, 244). Together, these studies support reciprocal immune–endothelial amplification while indicating that the relevant adhesion molecules, chemokines, and proteases depend on model, time point, and tissue compartment.

As the immune response evolves, selected myeloid-cell states can limit inflammatory injury or support recovery, but these functions should not be reduced to a single repair-associated category. In mouse transient MCAO, IL-33/ST2 signaling stimulated microglial IL-10 production and limited acute ischemic injury. MANF was induced in microglia/macrophages in human and rodent ischemic tissue, and recombinant MANF reduced infarct size and inflammatory cytokines in rat stroke, although direct vascular-repair effects remain unproven (245, 246). In male mice, microglia/macrophage vitamin-D-receptor signaling restrained TNF-α/IFN-γ-driven endothelial CXCL10 release, BBB disruption, and T-cell infiltration (247). CNS-border-associated CD163+ macrophages can also be injurious: in rat transient MCAO, depletion and transcriptomic analyses linked them to granulocyte recruitment, VEGF expression, vascular leakage, and acute neurological impairment (248). Perivascular macrophages, brain-resident microglia, and endothelial cells communicate bidirectionally to regulate barrier homeostasis and damaged-microvessel clearance, but their net effects depend on cell state and phase (249, 250).

The metabolic state of the local and systemic environment further shapes endothelial–immune interactions. Hyperglycemia can alter the endothelial secretome and cellular phenotype, increase BBB permeability, and modulate pericyte-derived chemokine release, thereby changing the pattern of immune-cell migration (251). Oxidative stress and inflammatory signaling generated by the vascular endothelium form reciprocal feedback loops with peripheral immune activation, creating complex spatial and temporal dynamics of BBB disruption. Thus, endothelial–immune crosstalk acts both as a driver of acute neurovascular injury and as a regulatory node for later vascular repair.

Thromboinflammation links hemostasis to post-ischemic immune and microvascular injury. Activated or injured endothelial cells release ultra-large von Willebrand factor (VWF) multimers, which capture platelets through glycoprotein Ib and promote platelet adhesion under high shear conditions. Platelet activation supports leukocyte recruitment through P-selectin/PSGL-1 and additional adhesive and chemokine signals, facilitating platelet–neutrophil aggregates, NET formation, and microvascular obstruction. The VWF–platelet glycoprotein Ib alpha (GPIbα) axis mediates ischemic brain injury that cannot be explained by infarct-artery thrombosis alone (252) and connects endothelial injury, coagulation, innate immunity, and no-reflow (253).

ADAM metallopeptidase with thrombospondin type 1 motif 13 (ADAMTS13) counter-regulates this pathway by cleaving ultra-large VWF multimers. Lower ADAMTS13 activity has been associated with increased risk of IS in human studies (254), whereas experimental enhancement of VWF cleavage can reduce thromboinflammatory injury. The clinical effects of manipulating VWF or ADAMTS13 may depend on recanalization treatment, bleeding risk, stroke etiology, and sampling time.

### 6.4 Stage-dependent immunometabolic transitions

After ischemic brain injury, microglia and infiltrating macrophages undergo heterogeneous, phase-dependent metabolic reprogramming rather than a uniform M1-to-M2 transition. Inflammation-associated states frequently show increased glycolytic and pentose phosphate pathway activity, altered glutamine utilization, and reduced mitochondrial flexibility, whereas selected phagocytic or repair-associated states can engage oxidative phosphorylation, fatty-acid oxidation, glutaminolysis, and lysosomal metabolism to support neutrophil or debris clearance and tissue remodeling (11, 255, 256). These associations are neither exclusive nor irreversible. Increased lactate production can also regulate inflammatory gene expression through histone H4 lysine 5 lactylation and H3K9la, linking metabolic flux to context-dependent immune effector functions (172, 257).

Specific metabolic checkpoints are best evaluated through defined functions rather than binary labels. Experimental studies have linked HK2-dependent hyperglycolysis and CKLF1-related metabolic reprogramming to microglial inflammatory output after experimental stroke (258, 259). TPI1, SPTLC2, and PKM2 have each been linked to glycolytic control, lipid handling, inflammatory-mediator production, or phagocytosis in experimental stroke (260–262), but their effects remain model- and context-dependent. Therapeutic value will require cell-resolved target engagement and validation beyond single experimental systems.

During the subacute and chronic phases, selected phagocytic or repair-associated subsets show increased lysosomal, mitochondrial, and lipid-handling programs, but a global shift of all microglia and macrophages toward fatty-acid oxidation and oxidative phosphorylation has not been demonstrated. CD11c+ microglia expressed phagocytic, myelin-supportive, and lipid-metabolism programs during white-matter recovery (104), whereas GPNMB-high repair-associated MDMs displayed enhanced lipid metabolism and proangiogenic activity after early microglial attenuation (164). These observations support cell-state-resolved metabolic checkpoints rather than a binary polarization model.

### 6.5 Brain-border influx–efflux coupling

The brain-border system extends the interaction network beyond the conventional NVU. Local influx can originate from skull marrow niches and be shaped by meningeal mast cells, nociceptive signals, stromal cues, and dural vascular interfaces. Efflux through meningeal lymphatics simultaneously transports fluid, soluble mediators, antigens, and immune cells toward cervical lymph nodes. Stroke outcome may therefore depend on an influx–efflux balance rather than on leukocyte entry alone: local immune recruitment, parenchymal clearance, cerebrospinal fluid drainage, and lymph node activation can occur in parallel and can have opposing effects.

This balance is tightly coupled to vascular and metabolic status. Edema changes interstitial pressure and convective flow; endothelial and pericyte injury alters entry routes; cell death changes the cargo presented to border immune cells; and systemic metabolic disease may modify marrow output and lymphatic function. A useful experimental unit is therefore not an isolated inflammatory mediator but a defined compartmental circuit—for example, ischemic tissue to cerebrospinal fluid, skull marrow or dura, and cervical lymph node—measured over time with both recruitment and clearance readouts.

## 7 Stage-specific therapeutic strategies targeting neurovascular, immune, and metabolic remodeling

Reperfusion remains the foundation of acute ischemic stroke treatment. Intravenous thrombolysis and mechanical thrombectomy restore upstream arterial patency but also alter downstream thromboinflammation, BBB stress, oxidative injury, and metabolic demand. Standard secondary prevention and rehabilitation measures, including mechanism-specific antithrombotic therapy, statin therapy, vascular risk factor control, infection prevention and treatment, nutritional support, and task-specific rehabilitation, also shape the inflammatory and metabolic milieu. Experimental adjuncts should therefore be evaluated on top of contemporary standard care rather than against untreated ischemia alone.

### 7.1 Hyperacute phase: recanalization, thromboinflammation, and microvascular reperfusion

The hyperacute therapeutic priority is rapid and safe reperfusion. Intravenous thrombolysis and mechanical thrombectomy restore macrovascular patency in eligible patients, whereas tissue-level recovery also depends on collateral flow and downstream microvascular reperfusion. Adjunctive targets include endothelial activation, VWF-dependent platelet adhesion, platelet–leukocyte aggregates, NET formation, pericyte contraction, endothelial swelling, and neutrophil capillary stalling. Because these same pathways contribute to hemostasis and vascular integrity, any adjunct to reperfusion must be evaluated for intracranial bleeding and should not be presented as a substitute for established recanalization therapy.

Although large-vessel recanalization remains the primary goal of endovascular therapy for acute IS, increasing evidence indicates that macrovascular reopening alone is insufficient to restore cerebral tissue perfusion fully. Preservation of microcirculatory homeostasis is therefore essential for downstream tissue protection. In this context, microcirculatory homeostasis refers to the dynamic balance among cerebral microvascular perfusion, capillary permeability, endothelial and pericyte function, BBB integrity, and local metabolic and oxygen demands. This balance ensures adequate oxygen delivery to the ischemic penumbra while limiting cerebral edema and inflammatory propagation (5, 263).

During the acute phase, pericyte contraction and microvascular obstruction are key mechanisms of no-reflow. Human pluripotent stem cell-derived pericyte-like cells retained vascular markers and selected functions under *in-vitro* hypoxia, but this work does not establish an effective post-stroke cell therapy (264). In a rat MCAO/reperfusion model, tenecteplase administered before reperfusion reduced CD31^+^/fibrinogen^+^ and CD31^+^/thrombocyte^+^ microvascular deposits and improved downstream perfusion (265). These experiments provide mechanistic support for microvascular reperfusion, but dosing, hemorrhagic risk, and added benefit beyond contemporary thrombectomy must be established clinically.

### 7.2 Acute phase: barrier protection, inflammatory amplification, and regulated cell death

During the acute phase, the therapeutic objective shifts toward limiting secondary injury without producing systemic immunosuppression. BBB disruption, mitochondrial dysfunction, oxidative stress, innate immune sensors, and regulated cell-death programs form an interconnected network. Potential interventions should be evaluated for target engagement in specific cells and for effects on edema, hemorrhagic transformation, infection, and neurological function. Evidence for ferroptosis, necroptosis, and pyroptosis is primarily preclinical, while evidence for cuproptosis in cerebral ischemia remains emerging; none should be described as a clinically established target.

Immune-inflammatory responses span the entire course of IS, but their effects depend on treatment timing, immune-cell subset, reperfusion state, and local tissue context. Thus, immunotherapy should modulate a defined injurious function rather than suppress inflammation globally. In mice subjected to 30 min of transient MCAO, delayed NLRP3 inhibition beginning 24 h after stroke reduced subacute infarct progression and improved function (266). Strategies directed at neutrophil recruitment, NETs, or astrocyte–neutrophil signaling remain predominantly preclinical and should be assessed for microvascular benefit, infection risk, and preservation of reparative functions (267).

Broad immunosuppression during the acute phase may reduce sterile inflammation but aggravate infection risk; therapeutic strategies should preserve host defense and be paired with infection-aware patient stratification.

### 7.3 Subacute phase: immune resolution, metabolic adaptation, angiogenesis, and white-matter repair

The subacute phase requires a transition from injury suppression to coordinated resolution and repair. Reparative strategies may promote efferocytosis and debris clearance, regulatory lymphocyte function, endothelial stabilization, angiogenesis, oligodendrocyte lineage recovery, remyelination, and restoration of mitochondrial and substrate flexibility. However, excessive or poorly timed angiogenic and immune stimulation may increase barrier permeability or maladaptive inflammation. Treatment should enhance defined repair functions rather than globally activating a cell type.

Immune responses gradually shift toward tissue clearance and repair during the subacute and chronic phases; continued broad immunosuppression may therefore impair debris clearance, vascular stabilization, and axonal remodeling. Microglia and infiltrating macrophages participate in necrotic cell and myelin debris clearance (33, 268). Representative intervention evidence remains model-specific: IL-10 mRNA-loaded, microglia-targeted lipid nanoparticles improved barrier and inflammatory outcomes in a mouse model of transient MCAO (269); P2X4R perturbation altered phagocytosis in genetically modified or inhibitor-treated mice (93); and anti-ferroptosis exosomes engineered to target a repair-associated microglial population (described as “M2” in the original study) were tested in MCAO mice (270). These platforms are mechanistic prototypes, not equivalent therapeutic candidates.

The time-window dependence of immune modulation is illustrated by complement C3a/C3aR signaling. In mice, acute C3aR inhibition and delayed intranasal C3a beginning on day 7 had opposing phase-specific effects, with delayed treatment modifying astrocyte reactivity and recovery (271). Separate experimental work linked microglial IL-1α, TNF, and C1q to reactive astrocyte programs after stroke (272). These studies support time-dependent biology but do not establish a human therapeutic window.

### 7.4 Chronic phase: persistent neuroinflammation, plasticity, and rehabilitation

Chronic treatment should address persistent neuroinflammation and network-level recovery. Delayed T- and B-cell responses, chronic microglial and astrocytic states, white-matter degeneration, impaired remyelination, and metabolic consequences of inactivity may influence cognition and disability for weeks to months. Pharmacological approaches should be integrated with rehabilitation, physical activity, sleep, nutrition, and control of vascular risk factors, using long-term functional and cognitive outcomes rather than infarct volume alone.

Peripheral adaptive immune cells also display strong stage- and subset-dependent effects. Tregs primarily attenuate neuronal injury during the acute phase by suppressing excessive inflammation, whereas during the chronic phase they may promote neurogenesis, tissue repair, and functional recovery. Potential therapeutic strategies include adoptive Treg transfer, pharmacological expansion, and mucosal tolerance induction. Nevertheless, their clinical application still requires careful optimization of therapeutic timing, dosing, and long-term efficacy (273).

Structural remodeling of the NVU depends on coordinated interactions among multiple cell types. In male Sprague–Dawley rats, ischemic postconditioning after transient MCAO increased astrocytic BDNF and was associated with angiogenic and neurogenic markers (274). This result is a model-specific proof of principle; it should not be generalized to a stand-alone BDNF therapy. Single-cell studies identify heterogeneous repair-associated astrocyte states, but their stability, human homologs, and tractability remain unresolved (275).

Microglia also participate in microvascular regeneration and tissue reconstruction. Nfat1-deficient and wild-type mouse experiments linked an NFAT1-positive microglial subset to HAS3–hyaluronan–LYVE1 signaling, anti-inflammatory polarization, and angiogenesis (276). Separately, dl-3-n-butylphthalide–vimentin–Notch signaling was examined in rat MCAO/reperfusion and cell models (277). Both findings require validation across sexes, ages, comorbidities, laboratories, and reperfusion regimens before they can support stage-specific clinical recommendations.

### 7.5 Cross-stage translation: standard care, combination treatment, biomarkers, and targeted delivery

Cross-stage translation requires integration with standard care and explicit evidence grading. The candidates discussed above span *in vitro* hypoxia, filament-induced transient MCAO, permanent or embolic occlusion, photothrombotic injury, genetically modified animals, nanoparticle delivery, and human observational or interventional evidence. These models answer different questions and should not be ranked by effect size alone. Unless a human trial is explicitly cited, the therapeutic claims in this chapter should be interpreted as preclinical. Combination therapy should pair a stage-matched adjunct with reperfusion, secondary prevention, or rehabilitation and should define sequence, duration, pharmacokinetics, target engagement, and safety interactions.

Consistent with contemporary Stroke Therapy Academic Industry Roundtable and multicenter preclinical network recommendations, a high-priority candidate should demonstrate randomized and blinded efficacy with prespecified inclusion criteria; adequate power; clinically realistic post-onset dosing; pharmacokinetic and pharmacodynamic target engagement; compatibility with thrombolysis or thrombectomy; replication across laboratories and stroke models; inclusion of both sexes, aging, and relevant comorbidities; and assessment of hemorrhage, edema, infection, cognition, and long-term function (278, 279). Targeted nanoparticles, EVs, or other delivery systems may improve exposure, but a delivery platform cannot compensate for an inadequately validated target.

Clinical translation of anti-inflammatory and neuroprotective strategies has been difficult. The anti-ICAM-1 antibody enlimomab worsened outcomes in acute IS (280), the neutrophil-inhibitory agent UK-279,276 did not improve outcome in ASTIN (281), and trials of ebselen, abciximab (AbESTT-II), and high-dose albumin (ALIAS Part 2) were also neutral or harmful (282–284). This pattern and its mechanistic implications are synthesized in a recent review of thromboinflammatory translational barriers (287). SCIL-STROKE demonstrated pharmacodynamic activity rather than functional efficacy: anakinra significantly reduced IL-6 and C-reactive protein, but did not improve the modified Rankin Scale score (OR 0.67, 95% CI 0.29–1.52; P = 0.34) (285). More recent trials provide qualified positive signals. In IRIS, adjunctive tocilizumab during endovascular treatment reduced 72-h infarct-core growth (8.8 mL vs. 27.0 mL; adjusted difference in cubic-root volume −0.41, 95% CI −0.79 to −0.03; P = 0.04) and was associated with numerically fewer symptomatic intracranial hemorrhages (3/51 vs 7/57), but the phase 2 sample was small and a 90-day functional benefit was not established (288). In EMPHASIS, minocycline initiated within 72 h increased the rate of excellent 90-day outcome (mRS 0–1: 52.6% vs 47.4%; adjusted risk ratio 1.11, 95% CI 1.03–1.20; P = 0.0061) and favored the ordinal mRS shift, with similar overall safety (289). These findings warrant confirmation across broader populations, stroke severities, and reperfusion contexts before routine adoption.

**Figure 5:**
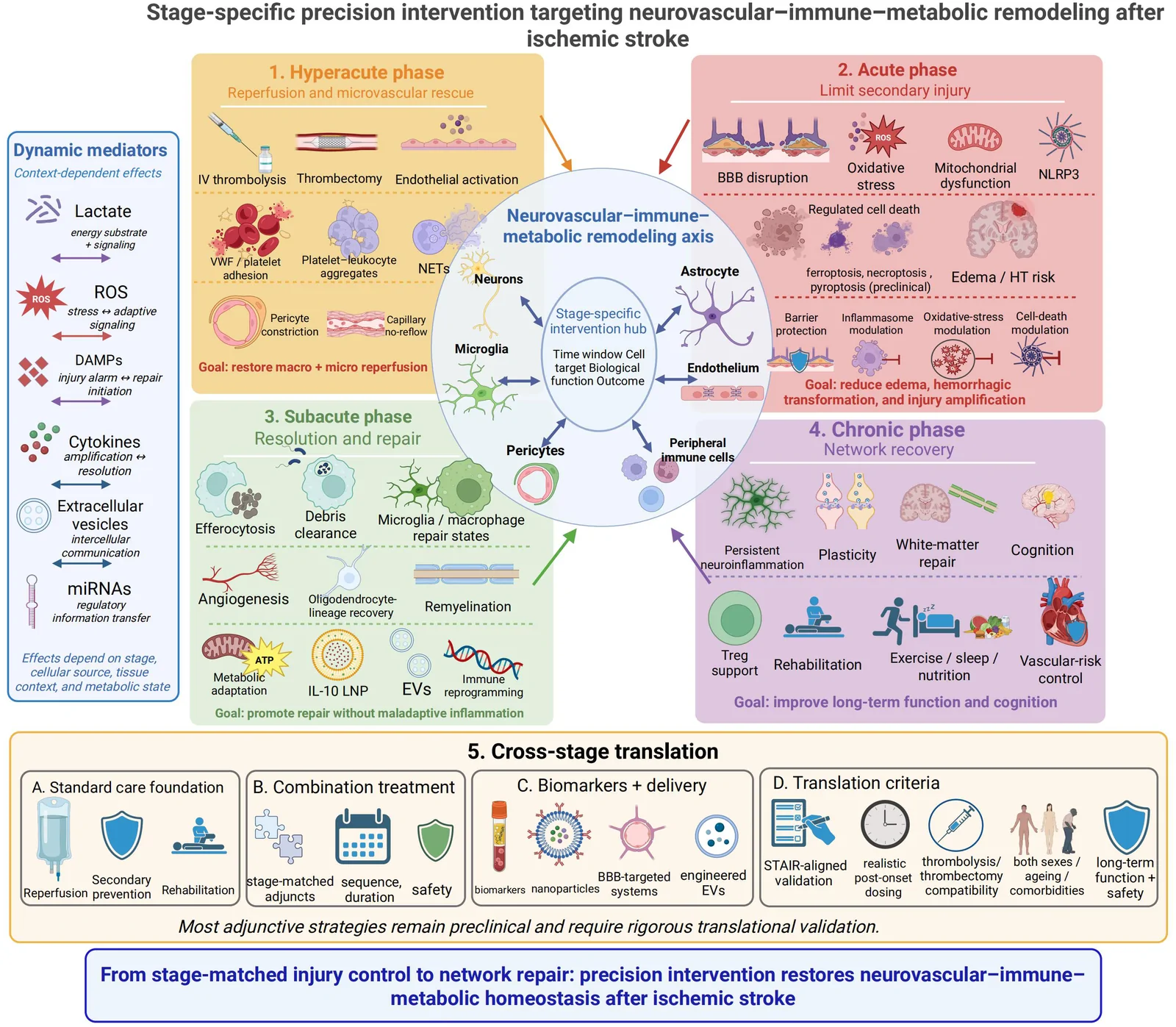
Stage-specific precision intervention targeting neurovascular–immune–metabolic remodeling after ischemic stroke. The central neurovascular–immune–metabolic remodeling axis connects neurons, astrocytes, microglia, endothelial cells, pericytes, and peripheral immune cells with a stage-specific intervention hub defined by treatment time window, cellular target, biological function, and outcome. In the hyperacute phase, the priority is reperfusion and microvascular rescue. IV thrombolysis and thrombectomy restore macrovascular flow, whereas endothelial activation, VWF/platelet adhesion, platelet–leukocyte aggregates, NETs, pericyte constriction, and capillary no-reflow identify downstream microvascular processes relevant to restoring both macrovascular and microvascular reperfusion. In the acute phase, the aim is to limit secondary injury associated with BBB disruption, oxidative stress, mitochondrial dysfunction, NLRP3-related inflammasome signaling, regulated cell death—including ferroptosis, necroptosis, and pyroptosis, which are marked as preclinical—and edema/HT risk. Barrier protection, inflammasome modulation, oxidative-stress modulation, and cell-death modulation are depicted in relation to the goal of reducing edema, hemorrhagic transformation, and injury amplification. The subacute phase shifts toward resolution and repair through efferocytosis, debris clearance, microglial/macrophage repair states, angiogenesis, oligodendrocyte-lineage recovery, remyelination, metabolic adaptation, IL-10 LNPs, EVs, and immune reprogramming, with the goal of promoting repair without maladaptive inflammation. The chronic phase emphasizes network recovery by linking persistent neuroinflammation, plasticity, white-matter repair, and cognition with Treg support, rehabilitation, exercise, sleep, nutrition, and vascular-risk control to improve long-term function and cognition. Cross-stage translation comprises four complementary layers: **(A)** a standard-care foundation consisting of reperfusion, secondary prevention, and rehabilitation; **(B)** combination treatment addressing stage-matched adjuncts, treatment sequence and duration, and safety; **(C)** biomarkers and delivery, including biomarkers, nanoparticles, BBB-targeted systems, and engineered EVs; and **(D)** translation criteria comprising STAIR-aligned validation, realistic post-onset dosing, compatibility with thrombolysis and thrombectomy, inclusion of both sexes, ageing, and relevant comorbidities, and assessment of long-term function and safety. Most adjunctive strategies shown remain preclinical and require rigorous translational validation. Solid phase-colored arrows connect the four disease phases with the shared remodeling axis, whereas blue solid bidirectional arrows indicate reciprocal coupling between the intervention hub and the indicated cellular compartments. In the dynamic-mediator key, horizontal double-headed arrows indicate context-dependent ranges of action rather than fixed harmful or reparative identities: lactate functions as an energy substrate and signaling molecule; ROS span stress and adaptive signaling; DAMPs span injury alarm and repair initiation; cytokines span inflammatory amplification and resolution; and EVs and miRNAs mediate intercellular communication and regulatory information transfer, respectively. These effects depend on disease stage, cellular source, tissue context, and metabolic state. The dashed horizontal lines within the phase panels are organizational separators and do not indicate causality or evidence grade; the blunt-ended red symbols in the acute intervention row denote modulation of the indicated pathways rather than a validated treatment effect. Green identifies microglia, consistent with the color convention used across the figure set. Together, the schematic depicts a therapeutic trajectory from stage-matched injury control to network repair and restoration of neurovascular–immune–metabolic homeostasis. ACOD1/IRG1, aconitate decarboxylase 1/immune-responsive gene 1; ATP, adenosine triphosphate; BBB, blood–brain barrier; BDNF, brain-derived neurotrophic factor; Ca^2+^, calcium ion; cfDNA, cell-free DNA; cGAS–STING, cyclic GMP–AMP synthase–stimulator of interferon genes; CH_3_, methyl group; CH25H, cholesterol 25-hydroxylase; Cu^2+^, copper(II) ion; DAMPs, damage-associated molecular patterns; dsDNA, double-stranded DNA; ECM, extracellular matrix; EVs, extracellular vesicles; Fe^2+^, ferrous ion; G6PD, glucose-6-phosphate dehydrogenase; GABA, γ-aminobutyric acid; GDNF, glial cell line-derived neurotrophic factor; GPX4, glutathione peroxidase 4; GSH, glutathione; HT, hemorrhagic transformation; IL, interleukin; IV, intravenous; LNPs, lipid nanoparticles; M1/M2, conventional labels for classically/alternatively activated myeloid states; miRNAs, microRNAs; MMP, matrix metalloproteinase; MMP-9, matrix metalloproteinase-9; mtDNA, mitochondrial DNA; NADPH, reduced nicotinamide adenine dinucleotide phosphate; NETs, neutrophil extracellular traps; NF-κB, nuclear factor kappa B; NK, natural killer; NLRP3, NOD-like receptor family pyrin domain-containing 3; NMDAR, N-methyl-D-aspartate receptor; NRF2, nuclear factor erythroid 2-related factor 2; O_2_•−, superoxide anion; OPC, oligodendrocyte precursor cell; OXPHOS, oxidative phosphorylation; PINK1, PTEN-induced kinase 1; PPAR, peroxisome proliferator-activated receptor; PPP, pentose phosphate pathway; ROS, reactive oxygen species; SDH, succinate dehydrogenase; STAIR, Stroke Therapy Academic Industry Roundtable; TCA, tricarboxylic acid cycle; TGF-β, transforming growth factor beta; TIMP-1, tissue inhibitor of metalloproteinases-1; TNF-α, tumor necrosis factor alpha; TREM2, triggering receptor expressed on myeloid cells 2; Treg, regulatory T cell; VWF, von Willebrand factor; ZO-1, zonula occludens-1; ΔΨm, mitochondrial membrane potential.

Brain-border and metabolic interventions illustrate why treatment timing must be built into target selection. VEGF-C-mediated lymphatic enhancement was beneficial when induced before experimental stroke but not when given acutely afterward (155), while other models found acute swelling or harmful lymph node chemotactic signaling associated with the same broader pathway (156, 157). Similarly, nicotinamide riboside rescue of aged monocyte dysfunction was demonstrated as pretreatment (98). Such data can establish mechanism and prevention potential, but they should not be described as evidence for post-onset rescue. Translation requires clinically realistic dosing after symptom onset, compatibility with thrombolysis or thrombectomy, and separate assessment of edema, infection, hemorrhage, and late repair (**Figure 5**).

## 8 Discussion

### 8.1 An expanded stage- and compartment-resolved model of IS

This review supports an inflammation-centered model in which IS evolves through interactions among five connected compartments: the occluded and reperfused intravascular space; the downstream microcirculation and NVU; the ischemic and peri-infarct parenchyma; brain-border tissues including meninges, skull marrow, cerebrospinal fluid, and cervical lymph nodes; and systemic immune–metabolic organs. Inflammation is neither a late complication nor an isolated leukocyte response. It is a coordinating process through which vascular obstruction, endothelial activation, mitochondrial stress, metabolic substrate changes, cell death, edema, and tissue repair influence one another. This broader spatial model helps reconcile why successful macrovascular recanalization may coexist with microvascular no-reflow, persistent tissue hypoxia, and ongoing inflammatory injury.

Time determines which links within this network dominate. During the hyperacute phase, thromboinflammation, endothelial swelling, pericyte contraction, platelet–leukocyte adhesion, and capillary stalling can limit the benefit of arterial reopening. Thromboinflammation does not end with recanalization: reperfusion can sustain platelet–leukocyte interactions, NET formation, endothelial injury, capillary obstruction, and BBB disruption, thereby contributing to ischemia–reperfusion injury during the early acute phase, as detailed in *Sections 2.2* and *6.3*. In the acute phase, leukocyte entry, innate immune sensing, regulated cell death, excitotoxicity, and redox stress intensify tissue injury, while systemic immunodepression begins to increase infection risk. During the subacute phase, glial and myeloid cells participate in debris clearance, angiogenesis, oligodendrocyte lineage responses, scar remodeling, and circuit stabilization. In the chronic phase, persistent white-matter inflammation, impaired metabolic support, and maladaptive vascular or immune remodeling may limit recovery or contribute to cognitive decline. These overlapping phases argue against static pathway labels and favor phase-dependent functional definitions.

Brain-border immunity adds an influx–efflux dimension that is absent from a blood-versus-brain framework. Skull and vertebral marrow can supply myeloid cells locally (149), while cerebrospinal fluid communicates directly with skull marrow niches (150). Meningeal lymphatics can support clearance, yet their manipulation has produced context-dependent effects across experimental stroke models (155, 157). The relevant therapeutic question is therefore not simply whether leukocyte entry or lymphatic drainage should be increased or decreased. It is which cargo and cell states move through a defined route at a defined stage, and whether the net result is harmful recruitment, beneficial clearance, or altered peripheral immune priming.

### 8.2 From binary cell labels to functions, fluxes, and cellular origins

The accumulated evidence argues against organizing post-stroke inflammation around fixed M1/M2 microglial or macrophage labels, A1/A2 astrocyte labels, or a single harmful–protective axis. Single-cell studies identify mixed, spatially restricted, and time-dependent states, while experiments that include both sexes do not necessarily find global sexual dimorphism in every microglial response (160). Functional measurements are more informative: phagocytosis of viable cells versus debris, efferocytosis, cytokine production, antigen presentation, extracellular trap formation, myelin clearance, trophic support, and regulation of endothelial integrity. Cellular origin is equally important because resident microglia, circulating monocytes, skull marrow-derived myeloid cells, and meningeal macrophages may converge morphologically while retaining distinct developmental histories and tissue instructions.

Metabolic measurements should likewise move from concentration snapshots to cell-resolved flux and function. Lactate may reflect severe glycolytic stress, astrocyte–neuron substrate transfer, or immune activation, depending on localization and oxygenation. Succinate can act as an inflammatory signal through HIF-1α and IL-1β (220) and can drive reperfusion-associated mitochondrial oxidant production after ischemic accumulation (221). Itaconate can counter-regulate macrophage metabolism through succinate dehydrogenase and NRF2-linked mechanisms (224, 225), whereas age-associated NAD+ depletion may prime monocytes toward hyperinflammation (98). These pathways show that metabolites are signaling intermediates that connect energy status to immune effector programs; they are not merely passive biomarkers.

A similar caution applies to regulated cell death. Ferroptosis, necroptosis, pyroptosis, apoptosis, and emerging copper-dependent mechanisms share upstream stressors and may interact. Early post-reperfusion ferroptotic and necroptotic signals can be partly cross-regulated by iron and pathway-selective inhibitors (210). PANoptosis is useful as a hypothesis for coordinated inflammatory cell death, but the co-expression of several markers cannot establish a unified execution complex or prove that one cell simultaneously completed multiple death programs. Progress will require temporal ordering, cell-specific perturbation, and direct comparison of single-pathway with combined interventions.

### 8.3 Human evidence: what is directly observed and what remains inferred

Human evidence is expanding but remains uneven across compartments and stages. Thrombectomy enables paired intracranial blood, systemic blood, and thrombus sampling during the hyperacute phase, providing direct access to local thromboinflammatory signals in large-vessel occlusion. Spatial analysis of retrieved thrombi suggests that large-artery atherosclerotic and cardioembolic clots can contain different myeloid programs (145). Human peri-infarct tissue obtained during decompressive craniectomy can reveal glial responses that cannot be inferred from blood, and recent single-nucleus sequencing identified astrocyte and oligodendrocyte lineage alterations within approximately 24 h (144). These advances provide essential anchors for translation.

Each source also has a characteristic bias. Thrombectomy cohorts preferentially sample severe anterior-circulation large-vessel occlusion after variable exposure to thrombolysis, anesthesia, devices, and reperfusion. A thrombus records the biology of clot formation and organization but does not reproduce the infarcted parenchyma. Intracranial blood reflects the vascular compartment at one procedural time point. Surgically resected human brain tissue is rare, clinically selected, regionally constrained, and may require imperfect comparator tissue. Peripheral blood is scalable and longitudinal but mixes stroke-driven signals with infection, stress responses, comorbidities, and treatment effects. These limitations do not diminish the value of human data; they define the questions each specimen can answer.

Mechanistic claims should therefore be graded by triangulation. The strongest case arises when a pathway is observed in relevant human tissue or fluid, associates with a stage-specific clinical or imaging phenotype, and is causally modified in multiple clinically relevant experimental models. Human–rodent comparisons already show partial conservation but substantial heterogeneity in leukocyte timing and abundance (138). Age, sex, diabetes, atherosclerosis, infection susceptibility, stroke etiology, collateral status, infarct location, and reperfusion must be treated as biological variables rather than *post hoc* explanations for failed replication. The result should be a chain of evidence, not an assumption that a molecular signature in one compartment identifies the therapeutic mechanism in another.

### 8.4 Therapeutic translation: timing, combinations, and safety boundaries

Reperfusion remains the clinical foundation, so adjunctive therapies should be designed around—not apart from—thrombolysis and thrombectomy. A hyperacute adjunct should improve microvascular reperfusion or reduce reperfusion injury without delaying recanalization or increasing intracranial bleeding. An acute-phase immunometabolic intervention should limit barrier failure, edema, and regulated cell death while preserving antimicrobial defense. A subacute intervention should enhance resolution, debris clearance, angiogenesis, remyelination, or metabolic recovery without reopening the barrier or promoting maladaptive inflammation. Chronic treatment should be judged by cognition, white-matter integrity, participation, and rehabilitation-dependent recovery rather than by infarct volume alone.

Context-dependent pathways make pretreatment and post-treatment especially important to distinguish. Prophylactic VEGF-C-mediated lymphatic expansion was protective in one experimental model, but an acute injection was not (155); other studies associated lymphatic signaling with acute swelling or harmful brain–cervical-lymph-node communication (156, 157). Nicotinamide riboside improvement of age-associated monocyte dysfunction was also demonstrated as pretreatment (98). These studies identify potentially important mechanisms and prevention biology, but they do not yet establish a feasible rescue therapy after symptom onset. The clinically relevant experiment must administer treatment after a realistic delay and test it with reperfusion, antithrombotic therapy, and common comorbidities.

Prior clinical failures reinforce the need for explicit safety boundaries and have been critically contextualized within a recent thromboinflammation-focused translational framework (287). Enlimomab worsened outcome (280), and neutrophil-targeted UK-279,276 failed in ASTIN (281), illustrating how species-specific pharmacology, immune activation, infection risk, and mistimed suppression of beneficial functions can defeat plausible targets. SCIL-STROKE further showed a biomarker–outcome dissociation: anakinra reduced IL-6 and C-reactive protein but did not improve mRS outcome (OR 0.67, 95% CI 0.29–1.52; P = 0.34) (285). Conversely, IRIS and EMPHASIS provide encouraging but not definitive evidence for stage-matched immunomodulation: tocilizumab reduced early infarct-core growth after endovascular treatment without establishing functional efficacy (288), whereas minocycline improved 90-day outcomes in EMPHASIS but requires confirmation in broader populations and reperfusion-defined subgroups (289). Future trials should therefore use stage-specific inclusion criteria and pharmacodynamic biomarkers but should also be powered for prespecified clinical outcomes. Infection, hemorrhagic transformation, vascular integrity, cognitive function, disability, and quality of life should be monitored across extended follow-up.

### 8.5 Priorities for mechanistic and translational research

A first priority is longitudinal, multi-compartment sampling. Where feasible, the same patient should contribute serial systemic blood, imaging, and outcome data, with intracranial blood and thrombus collected during thrombectomy and cerebrospinal fluid or tissue analyzed only when clinically indicated. Sampling time should be reported relative to symptom onset, recanalization, reperfusion, infection, and major interventions. Integrated single-cell, spatial-transcriptomic, proteomic, and metabolomic analyses can then distinguish local pathways from systemic correlates and connect molecular states to edema, no-reflow, hemorrhagic transformation, white-matter injury, and recovery.

A second priority is experimental realism and causal validation. Key findings should be reproduced in transient and permanent occlusion, with and without thrombolysis or thrombectomy-like reperfusion, and in aged animals of both sexes with clinically relevant diabetes, hypertension, or atherosclerosis. The aged-microglial Ifi27l2a response (159) and sex-specific TNFR2 effects on remyelinating microglia (161) illustrate why host context may expose mechanisms that young, single-sex models miss. Cross-species validation should test homologous cell states and functions rather than rely on matching marker names alone.

A third priority is a minimum evidentiary standard for emerging targets. For ferroptosis, necroptosis, pyroptosis, cuproptosis, or PANoptosis, studies should identify the affected cell type, demonstrate pathway dependence with genetic or orthogonal perturbations, define the therapeutic window, and measure interactions with other death programs. For brain-border targets, experiments should quantify both immune influx and fluid, antigen, or cell efflux. For metabolic targets, isotope tracing or other flux measurements should complement static metabolite concentrations. Only after mechanism, timing, target engagement, and safety are linked should a delivery platform or combination strategy be prioritized for clinical translation.

Finally, biomarker development should focus on actionable states rather than isolated associations. C-reactive protein is prognostic but nonspecific (286); cfDNA is source-heterogeneous; and circulating miRNAs can vary with cell origin, extracellular-vesicle isolation, hemolysis, treatment, and sampling time. A usable precision panel should combine onset time, reperfusion status, stroke etiology, vascular and barrier imaging, immune-cell phenotypes, metabolites, and evidence of organ complications such as infection or gut-barrier dysfunction. Biomarkers should be chosen because they identify a targetable mechanism, confirm drug engagement, or forecast a safety trade-off, and they should be validated separately across etiologic subtypes.

## 9 Conclusion

Ischemic stroke is a dynamic, multicompartment disease in which inflammation links arterial occlusion and reperfusion to microvascular no-reflow, NVU failure, immune-cell redistribution, metabolic signaling, regulated cell death, and tissue repair. The dominant mechanisms change from the hyperacute through the acute, subacute, and chronic phases; therefore, the same pathway may amplify injury at one time, support clearance or repair at another, or exert different effects across compartments.

The most credible translational path is not indiscriminate anti-inflammation but stage-matched and compartment-resolved modulation built on reperfusion, secondary prevention, and rehabilitation. Human intracranial and tissue studies should anchor target selection, longitudinal biomarkers should identify actionable states, and experimental models incorporating aging, both sexes, comorbidities, and clinically realistic reperfusion should define causal windows and safety. Within this framework, inflammation serves as the organizing interface of neurovascular, immune, and metabolic remodeling and provides a rationale for precision combinations that protect early tissue without compromising host defense, debris clearance, remyelination, or late neurological recovery.
